# A model of how depth facilitates scene-relative object motion perception

**DOI:** 10.1371/journal.pcbi.1007397

**Published:** 2019-11-14

**Authors:** Oliver W. Layton, D. C. Niehorster

**Affiliations:** 1 Department of Computer Science, Colby College, Waterville, Maine, United States of America; 2 Lund University Humanities Laboratory and Department of Psychology, Lund University, Lund, Sweden; Uppsala Universitet, SWEDEN

## Abstract

Many everyday interactions with moving objects benefit from an accurate perception of their movement. Self-motion, however, complicates object motion perception because it generates a global pattern of motion on the observer’s retina and radically influences an object’s retinal motion. There is strong evidence that the brain compensates by suppressing the retinal motion due to self-motion, however, this requires estimates of depth relative to the object—otherwise the appropriate self-motion component to remove cannot be determined. The underlying neural mechanisms are unknown, but neurons in brain areas MT and MST may contribute given their sensitivity to motion parallax and depth through joint direction, speed, and disparity tuning. We developed a neural model to investigate whether cells in areas MT and MST with well-established neurophysiological properties can account for human object motion judgments during self-motion. We tested the model by comparing simulated object motion signals to human object motion judgments in environments with monocular, binocular, and ambiguous depth. Our simulations show how precise depth information, such as that from binocular disparity, may improve estimates of the retinal motion pattern due the self-motion through increased selectivity among units that respond to the global self-motion pattern. The enhanced self-motion estimates emerged from recurrent feedback connections in MST and allowed the model to better suppress the appropriate direction, speed, and disparity signals from the object’s retinal motion, improving the accuracy of the object’s movement direction represented by motion signals.

## Author summary

Research has shown that the accuracy with which humans perceive object motion during self-motion improves in the presence of stereo cues. Using a neural modelling approach, we explore whether this finding can be explained through improved estimation of the retinal motion induced by self-motion. Our results show that depth cues that provide information about scene structure may have a large effect on the specificity with which the neural mechanisms for motion perception represent the visual self-motion signal. This in turn enables effective removal of the retinal motion due to self-motion when the goal is to perceive object motion relative to the stationary world. These results reveal a hitherto unknown critical function of stereo tuning in the MT-MST complex, and shed important light on how the brain may recruit signals from upstream and downstream brain areas to simultaneously perceive self-motion and object motion.

## Introduction

**Fig 1:**
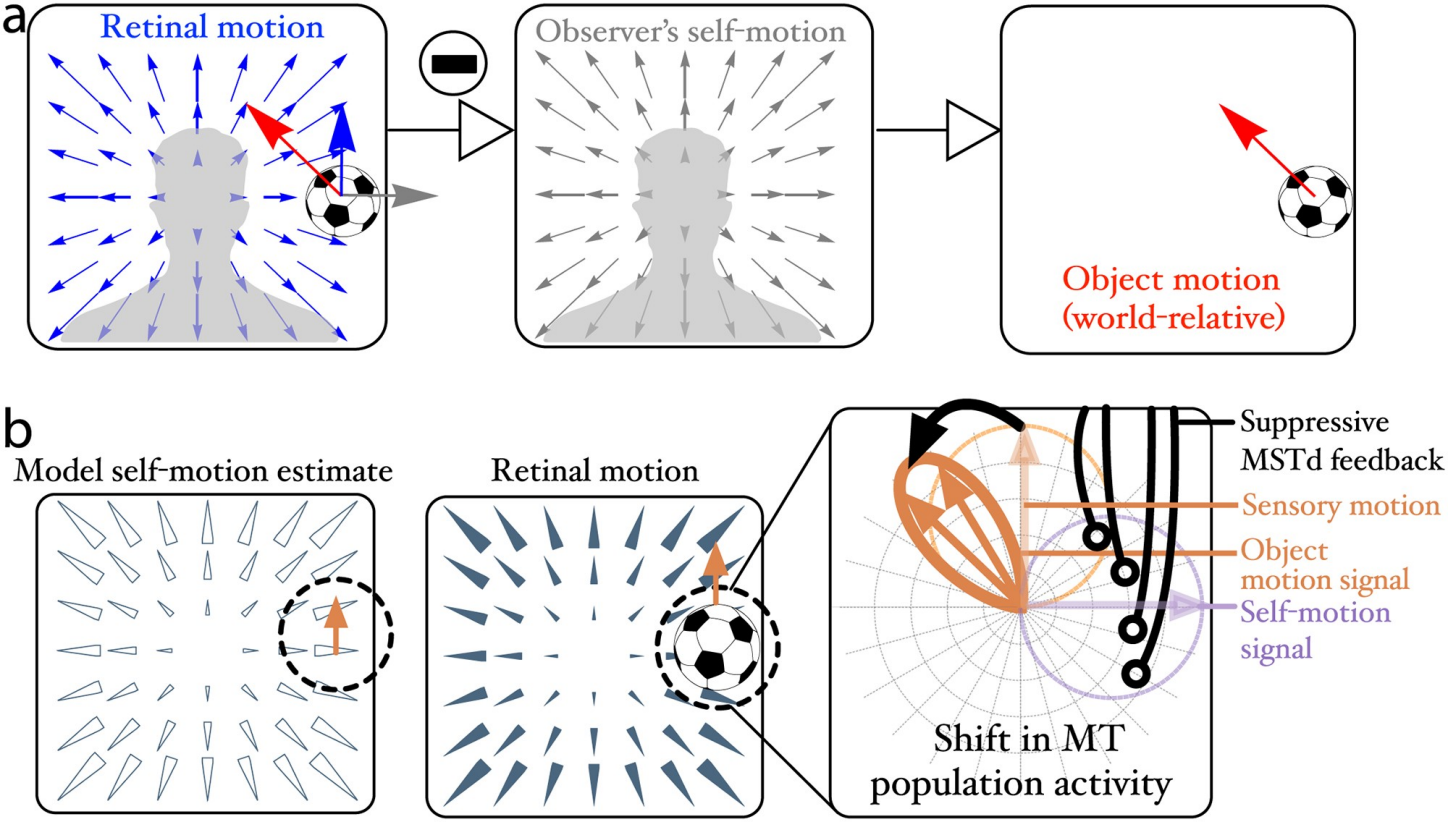
(a) Optic flow components on the retina of a mobile observer. The retinal motion (blue, left panel) is the sum of motion created through self-motion relative to world-fixed stationary environment (center panel) and the motion created by objects that move independently from the observer (red, right panel). The visual system could recover the world-relative motion of objects (red arrow, left and right panels), by subtracting the self-motion component (center panel) from the retinal pattern (left panel). (b) Neural algorithm proposed by Layton & Fajen [4] to recover world-relative object motion. The call-out on the right is a polar plot showing direction responses to the moving object. MSTd cells that respond to the observer’s self-motion send feedback to suppress MT cells (light blue region, right panel) that signal the retinal motion (light orange region, right panel) consistent with the preferred MSTd tuning (open arrows, left panel). Suppression shifts the direction signaled by the MT population toward the world-relative direction (dark orange, right panel).

It is a challenging problem for a moving observer to correctly perceive the movement of a moving object because the observer’s self-motion influences the retinal motion of the object (Fig 1a). Human psychophysical studies [1, 2] have provided strong evidence that the visual system solves this problem by attempting to remove the retinal component of visual motion that is due to self-motion. This suggests that the visual system transforms the retinal motion signal—the motion of the object in an observer-relative reference frame—into one fixed relative to the stationary world (i.e. world-relative). Not much is yet known about this process, however, as the neural basis of this hypothesis is just beginning to be explored [3, 4]. Preliminary evidence shows suppression and direction modulation when receptive fields are surrounded by the type of radial motion experienced by a moving observer, consistent with a world-relative shift [3].

To correctly factor out the observer’s self-motion from the object’s retinal motion and accurately perceive world-relative object motion, the visual system must account for the object’s depth. The retinal motion experienced by a moving observer is subject to motion parallax, which follows an inverse relationship between an object’s retinal speed and depth: observer movement relative to a faraway stationary object fixed in the world will generate a slower retinal motion than if the object were closer [5, 6]. Therefore, if the visual system incorrectly assesses depth, it will wrongly infer the proportion of retinal motion that arises due to self-motion. This would result in factoring out too much or too little of the retinal motion due the observer’s self-motion from the retinal object motion, potentially leading to errors in the perceived movement trajectory of the object.

The visual system estimates depth through monocular (e.g. pictorial) and binocular (e.g. disparity) cues [6]. Considering the importance of depth for accurate world-relative object motion perception, the quality of depth estimation from these cues should influence human object motion judgments during simulated self-motion. For example, adding monocular linear perspective to the visual scene has been shown to induce a large shift away from the retinal trajectory in judgments about an object’s movement direction [7]. Monocular cues alone, however, are unlikely to in general yield accurate estimates about depth because they only provide information about relative rather than absolute depth. Binocular disparity on the other hand offers quantitative, absolute depth information [8], which may support accurate object motion perception [9]. A visual solution, even with binocular disparity, however, may be insufficient: human object motion judgments under stereo viewing conditions approach, but do not reach ideal levels of accuracy where the observer would perceive world-relative object motion, without the influence of self-motion [2, 10–12].

In principle, there are two ways in which monocular and binocular sources of depth information could facilitate the accurate recovery of world-relative object motion: improved localization of the object’s depth relative to the observer (hereafter “improved depth localization”) and improved estimates of the retinal motion pattern (hereafter “global flow”) that arises from to the observer’s self-motion (hereafter “improved global flow estimation”). In the present study, we use computational modeling to propose plausible neural mechanisms for the latter, improved global flow estimation. The aim is to explain how depth information in the visual environment may influence estimates of the global flow to support accurate object motion judgments.

### Neural mechanisms of world-relative object motion perception

The hypothesis that improved global flow estimation could facilitate object motion perception is consistent with the Competitive Dynamics Model of Layton & Fajen [13] that specifies neural mechanisms upon which the visual system could rely to factor out an estimate of the observer’s self-motion from the neural motion signal (Fig 1b). The model includes interactions between MT neurons, well-known to respond to the motion of small moving objects [14], and neurons in dorsal MST (MSTd) shown to be causally related to self-motion perception [15, 16]. By the model’s account, improved global flow estimation would result from enhanced responses in MSTd, which implies improved sensitivity to the observer’s self-motion. Through the *MT*–MSTd interactions, the visual system may then better suppress the self-motion component from the object’s retinal motion and recover the world-relative motion of moving objects.

While the model of Layton & Fajen in its original form [4] did not specifically address depth, units within the elaborated version that we develop here exhibit joint tuning to motion parallax and disparity based on recent neurophysiological findings in MT and MST [9, 17–20]. This allows the model to account for the effect of depth structure of the scene. The model mechanisms raise the exciting possibility that MT and MST may combine estimates about the observer’s self-motion and depth to facilitate the perception of an object’s movement through a rigid and stationary world.

We started by exploring how neural units with well-established neurophysiological properties process object motion when there is uncertainty about depth in the visual scene (Simulation 1). Subsequent sections focus on how the same neural mechanisms process monocular (Simulation 2) and binocular (Simulation 3) motion parallax with increasingly precise depth information. In each case, we tightly coupled model simulations to match the conditions under which recent human data was collected where human subjects judged the trajectory of a moving object during simulated self-motion.

To anticipate, our simulations show how specific combinations of direction and speed gradients in the global motion parallax pattern may facilitate global flow estimation and world-relative object motion estimation through enhanced MSTd responses. In addition, we show how increases in the MT signal gain that occur in the presence of disparity [20] not only increase the gain in MSTd, but also increase pattern selectivity: competitive interactions generate more vigorous responses in the units tuned to the global motion pattern most compatible with the observer’s self-motion, while at the same time suppressing weaker responses in other units. This improves the efficiency with which MSTd-mediated feedback suppresses the self-motion component from object motion signals and thereby supports the more accurate perception of world-relative object motion.

## Materials and methods

Here we briefly review the common design and methodology shared among the human experiments on which we based the simulations that were performed to validate model mechanisms. In these experiments, humans made judgments of object motion perception while viewing simulated self-motion. These data have appeared previously in thesis [21] and publication [2] form. Furthermore, we provide an overview of the computational model of brain areas MT and MST and the protocol used in our simulations at the end of the section. Supporting Information S1 contains a complete mathematical specification of the neural model and explanation of the parameter values used throughout all simulations in this paper.

### Measuring object motion perception during self-motion: Motion nulling paradigm

The experimental data used for the simulations in this paper comes from multiple experiments [2, 21], which all measured object motion perception during self-motion using the same motion nulling paradigm and experimental logic.

The displays used in all the experiments were set up such that the retinal components due to object motion and due to self-motion were perpendicular to each other. For example, the moving object would be directly to the right of the focus of expansion (FOE) in the optic flow display such that the component of its retinal motion due to self-motion was rightward, while the object itself would move vertically, e.g., upward, creating a vertical retinal motion component due to object motion. These experiments all found that the flow parsing process removes only part of the retinal motion due to self-motion from the perceived movement of the object, and the residual self-motion component causes the object to be perceived as moving obliquely, in this example upward and to the right, away from the FOE.

The residual motion was measured through a motion nulling paradigm in which an additional motion component at various magnitudes toward or away from the FOE was added to the retinal motion of the object under the control of an adaptive staircase. By asking the participant to report whether the object moved obliquely leftward or rightward each trial, the experiments of Niehorster and colleagues [2, 21] determined the point of subjective equality at which the dot was perceived to move exactly in the object motion direction, thereby determining the magnitude of retinal motion due to self-motion that is not removed by flow parsing. Comparing this to the total retinal motion of the object due to self-motion yields a direct measurement of the accuracy of world-relative object motion judgments, which was called the *flow parsing gain*. A gain of 100% indicates that the visual system deducts 100% of the observer’s self-motion from the optical field of motion, effectively recovering world-relative object motion, whereas a gain of 0% indicates that none of the motion generated by the observer’s self-motion is factored out and the observer perceives the retinal motion direction. Furthermore, the angle at which the object is seen to move with respect to its true vertical motion direction is directly computed from the ratio of residual retinal motion due to self-motion and the retinal motion due to the object’s motion. Both these measurements of perceived object motion [2, 21] will be used in the simulations reported here.

### Computational model of MT and MST

#### Overview

**Fig 2:**
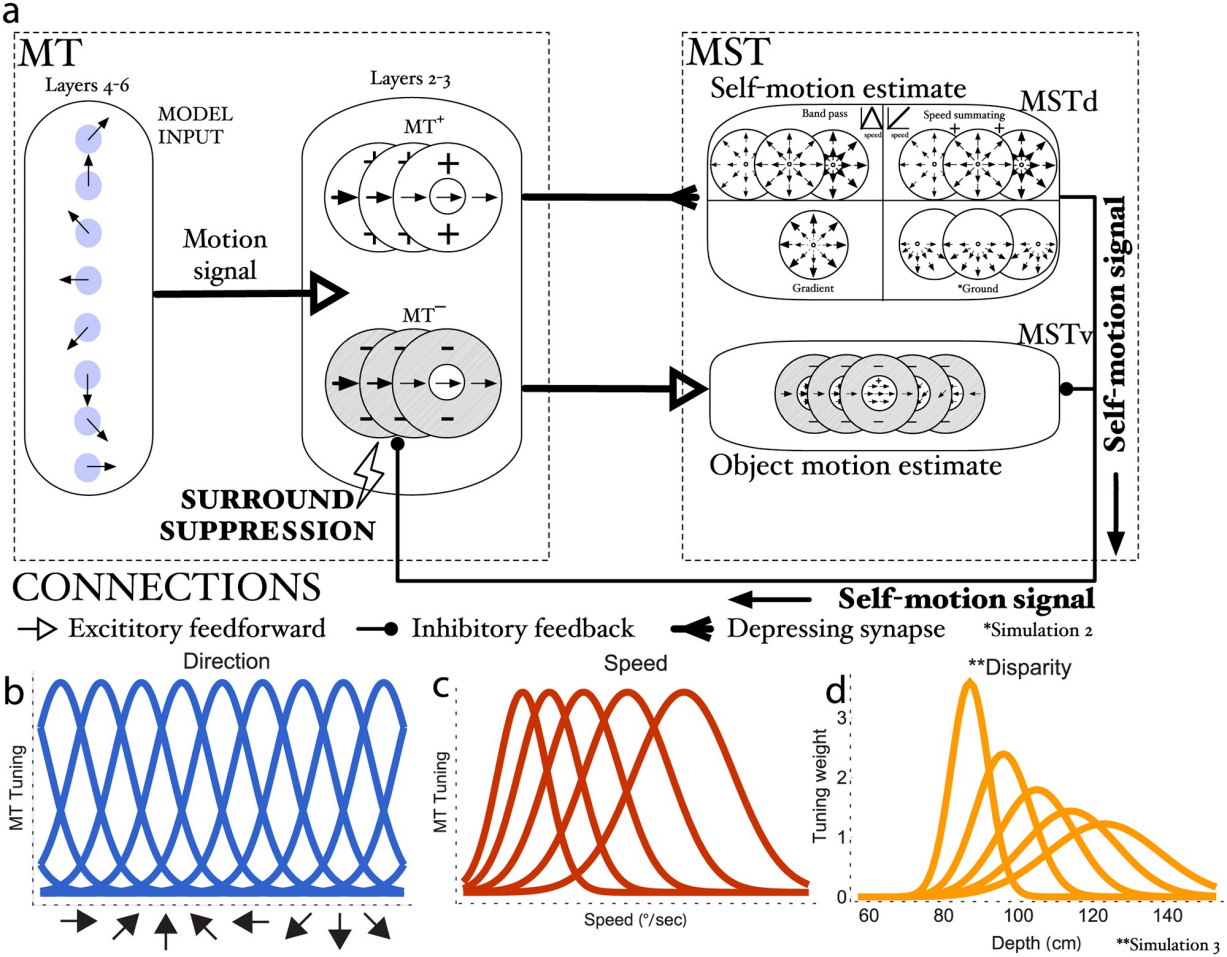
Diagram of model MT and MST. (a) The model contains two major pathways: *MT*^+^/MSTd subserves self-motion (top) and *MT*^−^/MSTv subserves object motion (bottom). The model is dynamic and estimates self-motion and object motion simultaneously. Cells in MSTd model physiologically supported populations tuned to full-field radial patterns, speed (band-pass, speed summating, gradient), and non-direction dependent disparities. Cells in *MT*^−^/MSTv have RFs with inhibitory surrounds due to local and feedback mediated suppression. Feedback comes from dominant MSTd units and suppresses *MT*^−^/MSTv units locally tuned to the direction, speed, and disparity that match the estimated self-motion motion pattern. This shifts population object motion responses from a retinal to world-relative reference frame. (b-d) Model MT tuning curves for direction (b), speed (c), and disparity (d). *MSTd units tuned to ground patterns were added in Simulation 2. **Disparity tuning was incorporated in Simulation 3.

Fig 2a shows an overview of the model. The model performs two main computational tasks: it estimates the pattern of motion that corresponds to the observer’s self-motion (model *MSTd*) and it signals the world-relative motion direction of moving objects (model *MT*^−^ and *MSTv*).

**MT L4, 6**. The input is represented by the vectors ${\vec{v}}_{x,y,f}$ specifying the motion (*v*_*dx*,*f*_, *v*_*dy*,*f*_) of points in the world at retinal position (*x*, *y*) and at frame *f* of the input sequence. This could be seen as the output of early visual processes that signal visual motion at a specific retinal location (e.g. LGN and V1). Direction and speed filters process the input signal and convert the motion vectors into a neural signal by Layer 4,6 MT units (hereafter referred to as MT L4,6); units respond to the input based on their tuning curves.

We show sample neural tuning curves for direction and speed in Fig 2b and 2c.

**MT^+^/MSTd pathway**. Units in the next stage, MT Layer 2-3 (hereafter referred to as MT L2-3), are jointly tuned to direction and speed, but process the output of MT L4,6 differently, depending on whether they possess reinforcing (*MT*^+^) or antagonistic (*MT*^−^) surrounds. This bifurcation reflects the anatomical segregation of MT into pathways that subserve self-motion and object motion [17, 18, 22]: *MT*^+^ units project to MSTd and *MT*^−^ projects to ventral MST (MSTv). *MT*^+^ units in the former pathway integrate the preferred direction and speed signals throughout their receptive fields. *MT*^+^ and MSTd units are connected by depressing synapses [23], which reduce the efficacy with which tonic *MT*^+^ signals activate MSTd neurons [24]. The resultant activity is matched against radial templates (Fig 2a; *MSTd*, top) that define the pattern selectivity of MSTd units. These templates implement the global motion pattern:

$$\begin{array}{c} \Pi_{i,j,x,y}=(x-i,y-j) \end{array}$$ (1)

where (*x*, *y*) indexes the regular spatial grid of MT receptive fields that span the visual field, and (*i*, *j*) corresponds to the preferred singularity position of the MSTd unit. We systematically varied (*i*, *j*) to generate MSTd cells tuned to different singular positions throughout the visual field, where (*i*, *j*) = (0, 0) corresponds to the center (e.g. Fig 1a). Although the global patterns span the entire visual field, we inversely weighted them by the distance from the singularity to define retinotopic receptive fields (Eq. S15, Supporting Information). This pattern selectivity combined with intra-areal recurrent dynamics allow MSTd units to estimate the visual pattern corresponding to the observer’s self-motion that takes both the speed gradient and radial pattern into account.

In all simulations, we model three subpopulations of radial (Eq 1) MSTd units tuned to different speed gradients: those that respond proportionally to retinal speed *speed summating cells* [25, 26], those that respond maximally to a particular average speed across the visual field *band-pass cells* [27], and those that respond to a speed gradient that grows with eccentricity *gradient cells* [27]. We model the speed sensitivity of speed summating cells as a weighted sum of *MT*^+^ speed signals:

$$\begin{array}{c} U(w_{s})=\frac{1}{\hat{s}}\sum_{s}sw_{s} \end{array}$$ (2)

where the function *U* integrates the activity of *MT*^+^ units tuned to speed *s* and the sum is normalized by the total number of preferred speeds ($\hat{s}$).

Band-pass cells inherit their sensitivity to average speed from *MT*^+^ units:

$$\begin{array}{c} B_{s}(w_{s})=w_{s}. \end{array}$$ (3)

Although band-pass cells only integrate cells maximally tuned to a single speed, their speed tuning curve makes them sensitive to a range of retinal speeds (Fig 2c).

Model gradient cells integrate signals from *MT*^+^ units tuned to different speeds depending on the eccentricity of the *MT*^+^ receptive fields. We include cells tuned to both increasing and decreasing speed gradients with eccentricity: gradient cells receive input from slow (fast) speed *MT*^+^ cells nearby the preferred singularity position and faster (slower) speeds at more distal positions.

As will be described, we introduced MSTd cells tuned to optic flow originating from a ground plane in Simulation 2 to account for differences in the experimental stimulus. Including these units in the frontoparallel plane simulations and in those from Simulation 3 where motion through a cloud was used had a weak effect on the model direction readout (object motion directions remained within ±1° in every case). We therefore omitted these ground units from Simulations 1 and 3 for simplicity.

To better account for the stereo viewing conditions in the displays used in Simulation 3, we add disparity tuning to model MT (Fig 2d) and MST.

**MT^−^/MSTv pathway**. Units in the *MT*^−^/*MSTv* pathway have inhibitory surrounds that are tuned to similar directions and speeds as the centers. Both areas perform an on-center/off-surround integration of motion and disparity signals, with the exception that MSTv units respond proportionally to retinal speed [28] like MSTd speed summating cells (Eq 2). Maximal surround suppression occurs when center and surround motion signals match (i.e. in a local region of uniform speed and direction). We read out the model’s estimate of the object’s motion, usually in area MSTv, from units that contain the moving object within the receptive field at the end of each simulation. We used a population estimate when computing model-derived estimates to compare with human judgments (see Section Direction readout).

**Fig 3:**
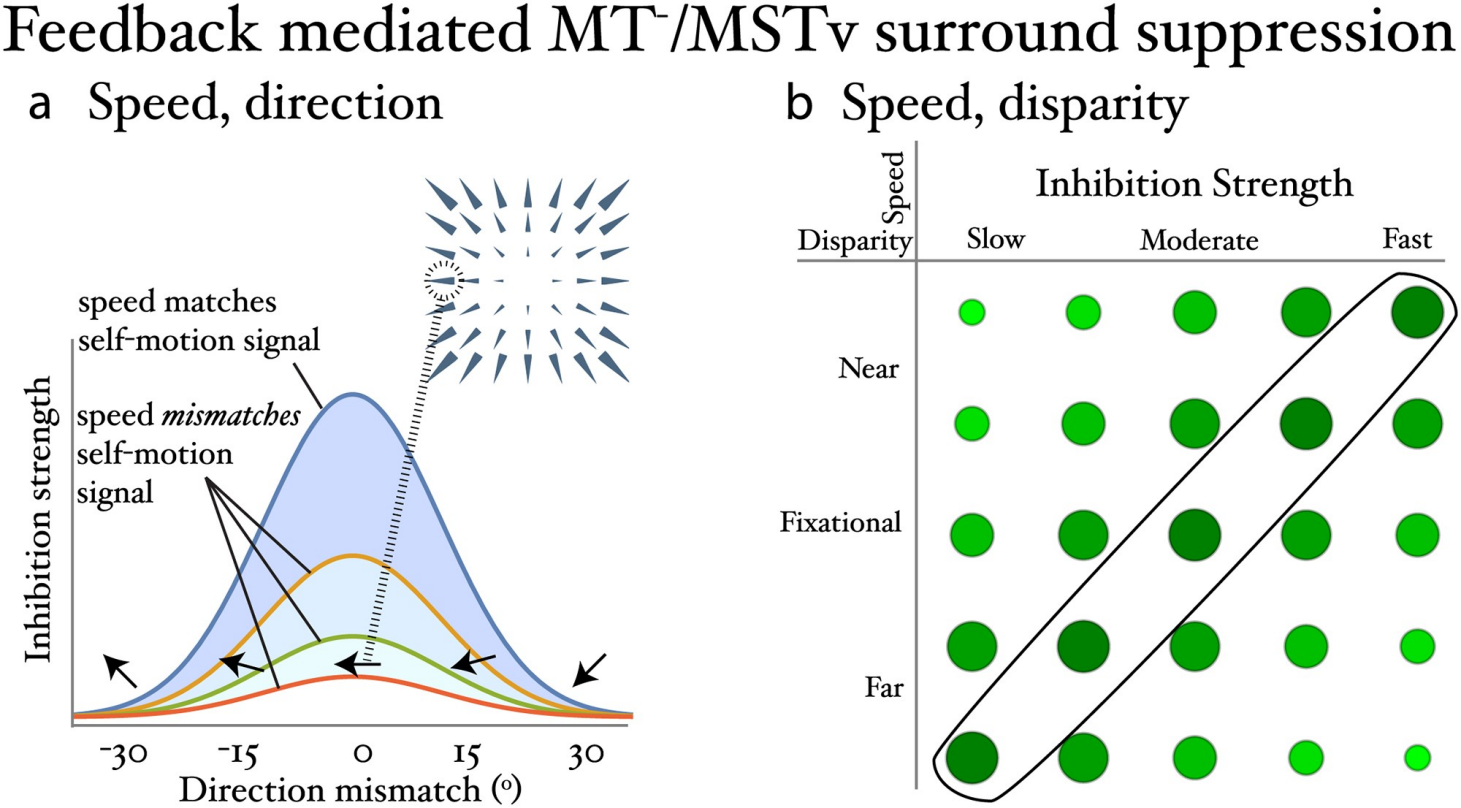
Overview of suppressive feedback mechanism. *MT*^−^ and MSTv units are inhibited depending on how their tuning properties relate to the corresponding local subregion of the most active MSTd unit’s RF. (a) The pattern selectivity of the MSTd unit is indicated on the top-right and the RF of some MT units are shown in left superimposed circle. Highest peak curve in plot shows that MT suppression is greatest when the MT direction tuning locally matches (leftward; 0° mismatch) the MSTd RF; units with mismatching direction tuning receive progressively less inhibition. Other curves show that inhibition drops off as speed tuning locally differs from the MSTd RF. (b) *MT*^−^ and MSTv units receive the most inhibition when they match the congruent speed-disparity tuning of the MSTd unit (circled diagonal). For example, among units with RFs near the FOE, those tuned to slow and far receive more inhibition than those tuned to slow and near.

The model contains feedback from MSTd units, that estimate the observer’s self-motion, to units in *MT*^−^ and MSTv. As Fig 3 depicts, the overarching rule is that MSTd feedback suppresses *MT*^−^/MSTv units that locally match the visual pattern to which the most active MSTd unit is tuned. Fig 3a shows that the suppression drops off as tuning in direction and/or speed becomes more dissimilar among units that share an overlapping receptive field (i.e. they occupy the same *MT*^−^ or *MSTv* macrocolumn). For example, suppression decreases in *MT*^−^/MSTv units with receptive fields that coincide with the left side of the horizon and deviate in speed (non-blue curves) and direction (positions along the blue curve that do not correspond to the maximum) tuning from the motion parallax pattern depicted in Fig 3a. We use the following Gaussian function to define this feedback weighting in speed or direction (*μ*):

$$\begin{array}{c} W\left(w;\mu ,\sigma \right)=e^{-{\left(\frac{w-\mu }{\sigma }\right)}^{2}} \end{array}$$ (4)

where *σ* refers to the rate at which the inhibition drops off as the local tuning of the *MT*^−^/MSTv direction and/or speed deviates from the motion parallax pattern corresponding to the observer’s estimated self-motion. The overall feedback signal is modulated by the activity of the most active MSTd unit.

#### Direction readout

To estimate the direction of a moving object from the model’s neural signals, we relied on a population vector readout technique whereby we weight the direction preference by the firing rate among units that respond to a moving object. With this approach, activity from the entire population of direction tuned units factor into the estimate [29]. This increases the precision of the object direction estimate compared to a winner-take-all approach because it is less constrained by angular quantization (15° in our simulations) in the range of peak tuning angles simulated in the model.

$$\begin{array}{c} \vec{J}(w_{d};\theta_{d})=\frac{1}{\sum_{d=1}^{\hat{d}}w_{d}}(J_{u},J_{v})=(\sum_{d=1}^{\hat{d}}w_{d}\cos\theta_{d},\sum_{d=1}^{\hat{d}}w_{d}\sin\theta_{d}) \end{array}$$ (5)

In Eq 5, *θ*_*d*_ refers to the direction tuning of the unit, *d* indexes the set of direction preferences, and $\hat{d}$ refers to the total number of direction preferences that subdivide 360°. Depending on the question of interest, we calculated the population vector for MSTv cells $\vec{J}(P_{x,y,d}^{v};\theta_{d})$ and occasionally for *MT*^−^ cells $\vec{J}(M_{x,y,d,s}^{-};\theta_{d})$. To extract the angle, we relied on the following equation:

$$\begin{array}{c} \bar{J}=\text{arctan2}(J_{v},J_{u}). \end{array}$$ (6)

Finally, we subtracted the object’s retinal direction *θ*_*Obj*_ from the model estimate to compute the shift or relative difference ${\overset{ˇ}{\theta }}_{Obj}$ which corresponds to the amount of retinal motion due to object motion that is removed by the model.

$$\begin{array}{c} {\overset{ˇ}{\theta }}_{Obj}=\bar{J}-\theta_{Obj} \end{array}$$ (7)

#### Simulations

We implemented the model in MATLAB and performed simulations using R2017a on a 4.3 Ghz quadcore desktop machine with 32GB of memory running Microsoft Windows 10. We numerically integrated the model using Euler’s method such that each new input frame was integrated for ten time steps. This amounts to a step size of ≈3 msec assuming 30 frames/s. Table 1 summarizes the number of units for each model area used in the simulations. RFs were uniformly distributed across the visual field, with the center of MSTd RFs sampling every fourth possible position of an MT cell in *x* and *y* (i.e., downscaled by 4x).

**Table 1:** Model unit counts within each area. *MSTd units tuned to ground patterns were added in Simulation 2. **Disparity tuning was incorporated in Simulation 3.

Area	Resolution	Count
MT+	64 (x) x 64 (y) x 24 (directions) x 5 (speeds) x 5** (disparities)	2457600
MSTd (band-pass)	16 (x) x 16 (y) x 5 (speeds) x 3** (disparities)	3840
MSTd (gradient)	16 (x) x 16 (y)	256
MSTd (ground)*	16 (x) x 16 (y)	256
MSTd (speed-summating)	16 (x) x 16 (y) x 3** (disparities)	768
MT-	64 (x) x 64 (y) x 24 (directions) x 5 (speeds) x 5** (disparities)	2457600
MSTv	64 (x) x 64 (y) x 24 (directions) x 5** (disparities)	491520

To generate plots of angle and flow parsing gain, we computed the analytic optic flow corresponding to the camera movement in each stimulus from the human experiments. We then discretized the motion to a $\hat{x}\times \hat{y}$ (64×64) grid (Table 1 in S1 Text), where MT cells RFs were centered on every pixel of the image. We presented the model with the resulting sequence of image frames. At the end of the sequence, we continued the simulation with the final motion vectors until model dynamics stabilized, defined as the population direction read out at the object remaining within 1° for a duration equivalent to at least five frames of video.

## Results

### Simulation 1: The influence of uncertainty in depth on the accuracy of object motion perception

Cells in primate MSTd demonstrate tuning to complex combinations of direction, speed, and disparity [27, 30, 31]. Given the potential importance of these cells in a neural mechanism for recovering world-relative object motion, as has been demonstrated by a previous version of the proposed model [4], we investigated the role that depth information serves in model MSTd dynamics. Specifically, we examined how uncertainty about the depth of stationary elements in the environment influences model MSTd activity and object motion signals. If depth information serves a central role, we expect viewing conditions that create a high degree of uncertainty about depth to only weakly stimulate model MSTd units. This should result in a modest influence on the retinal motion of a moving object (Fig 1b). That is, the model should fail to recover the world-relative motion because MSTd units can only suppress a small proportion of the retinal motion due to the observer’s self-motion from object motion signals.

**Fig 4:**
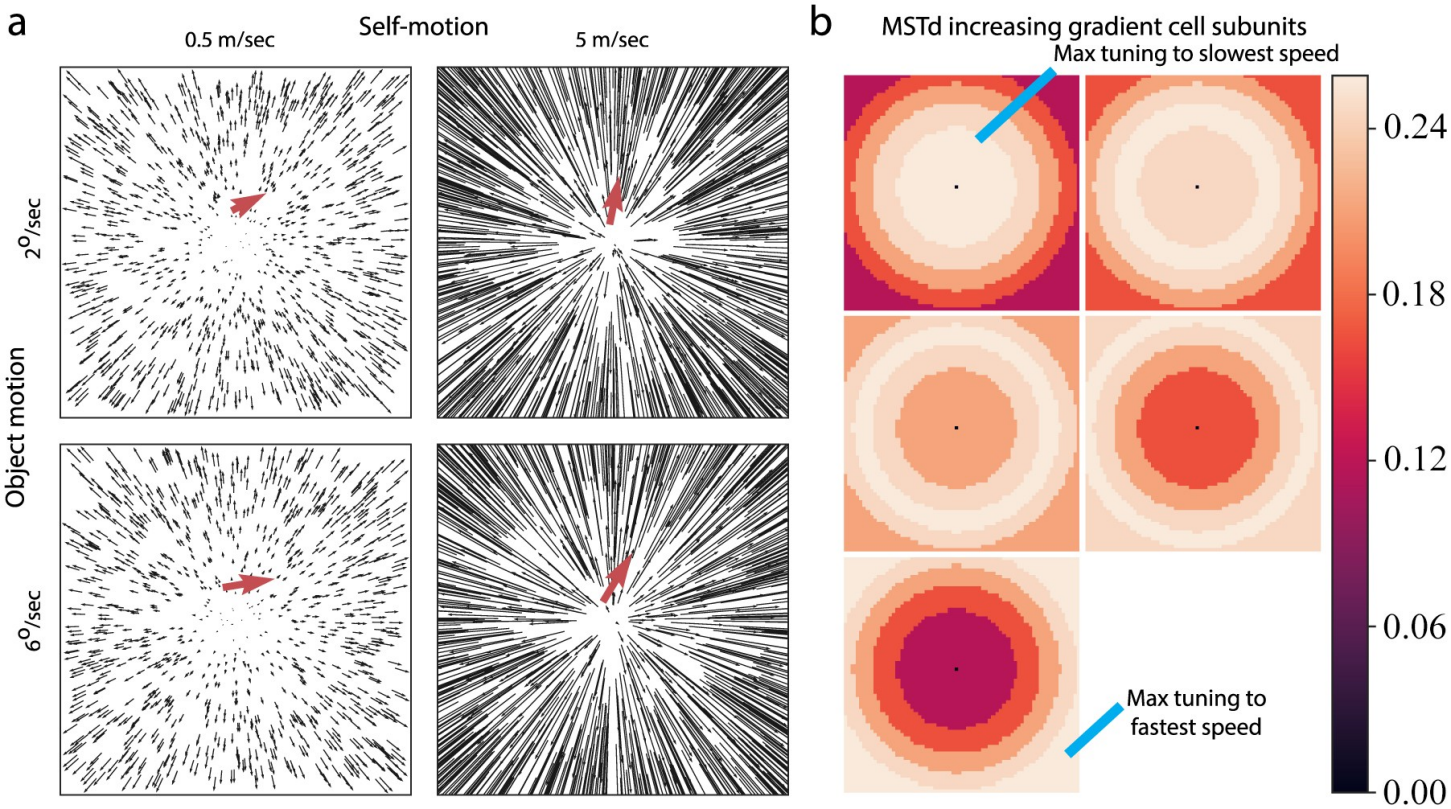
(a) Fronotoparallel displays used in Simulation 1. Optic flow depicting simulated self-motion at different rates of approach toward a frontoparallel plane of dots. The red arrow depicts the retinal motion of a small moving object. We scaled the length of the object vector 3x for visibility. (b) Model MSTd gradient cell RF subunits corresponding to sensitivity to the five different model MT peak speeds, which span the dynamic range across all frontoparallel stimuli (i.e. not necessarily any particular display). Increasing gradient cells prefer an increasing speed gradient and have the region of maximum sensitivity to the slowest (fastest) speed near the center (periphery) of the RF. The speed gradients in (a) stimulate a subset of the gradient cell annular subunit regions. Connection weights are depicted before normalization.

To create uncertainty about depth, we selected a monocular visual stimulus composed of dots arranged on a single frontoparallel plane. The monocular viewing and lack of depth variation prevent an observer from uniquely determining the depth of the plane. Fig 4a shows the optic flow produced by the stimulus as the simulated observer approaches the frontoparallel plane at 1°/s (left panels) and 10°/s (right panels). This creates an increasing speed gradient in the radial pattern that emanates from the FOE. A small object (diameter: 1°, motion indicated by the red arrow, Fig 4a) moved rightward on top of the frontoparallel plane (see next section for details).

The stimulus should recruit the MSTd gradient cell subunits (five panels in Fig 4b) that best match the increasing speed gradient at different eccentricities. For example, the 1 m/s self-motion condition (Fig 4a; top-left panel) may activate the center disk region of the MSTd subunit that prefers slowest speeds in the center (Fig 4b; top-left panel; bright disk). However, the peripheral motion may not be fast enough to strongly stimulate the subunit that prefers fast speeds in the outermost annular region (Fig 4b; bottom-left panel; bright region). This is because the dynamic range of image speeds in the 1 m/s condition is limited and the speed tuning range in each subunit corresponds to the range that may arise across all the frontoparallel plane stimuli, not necessarily any one display. Therefore, the stimuli should stimulate at least a subset of the MSTd gradient cell subunits.

The stimulus speed gradient should also activate speed summating cells considering that they integrate different speeds uniformly throughout the RF. However, like increasing gradient cells, the speed gradient should only excite a small portion of the total area of the RF.

Finally, model MSTd band-pass cells only respond to a particular MT speed band and should only be weakly activated by the gradient. Overall, we expect weak-to-moderate activity among the different MSTd cell subpopulations.

#### Simulation 1: Experimental data

We compared the extent to which model object motion signals reflected world-relative motion with human judgments using the same stimulus. [21, Chap. 6] has run a series of experiments examining the perception of world-relative motion during simulated self-motion toward a frontoparallel plane of dots. These stimuli were constructed such that the optical acceleration component due to approach of the plane was removed, yielding a field of constant retinal velocity throughout each trial, where velocity only depended on the distance of fiducial points to the focus of expansion.

A moving object was placed underneath the FOE of the flow field and had an object motion component that was directly leftward or rightward. To this object motion component a retinal motion component due to self-motion was added. The simulated self-motion speed was varied (0.5–5 m/s, corresponding to a self-motion component in the object’s retinal motion of 1–10°/s) to influence the direction of retinal motion of the object: faster self-motion speeds induce a larger shift in the retinal trajectory toward the self-motion component (Fig 4a). The object’s optical direction was also adjusted directly by setting its speed (2°/s or 6°/s). These stimuli were observed by static observers who made judgments about the trajectory of the moving probe object according to the procedure described in the methods section (see Retinal Motion Nulling in General Methods).

**Fig 5:**
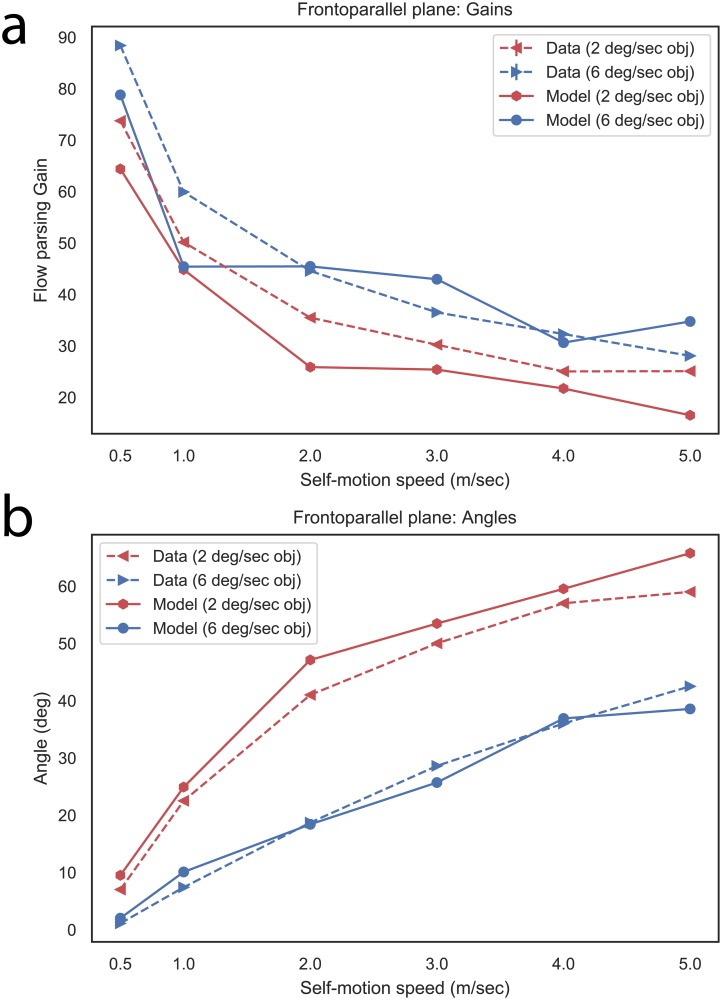
(a) The accuracy of human object motion judgments in Simulation 1 (frontoparallel plane), as measured by flow parsing gain (reproduced from Niehorster, 2013), compared with model object direction estimates. (b) Human judgments and model object direction estimates compared, expressed as angles. 0° indicates the retinal object direction and positive angles indicate shifts toward the world-relative direction.

Fig 5a summarizes human judgments about objects moving at 2 and 6°/s. In both cases, the flow parsing gain demonstrated a logarithmic decline, approaching gains of ≈0.30–0.40 at the faster self-motion speeds. The logarithmic decrease suggests that the visual system may be less efficient at deducting the global pattern at faster speeds. The faster object speed resulted in significantly higher gains. Fig 5b expresses these gains as angles to appreciate the magnitude of the shifts relative to the retinal object direction.

#### Simulation 1: Simulation results

Before addressing how the model responds to the uncertainty about depth in the frontoparallel plane stimulus, let us consider the case of forward translation along a central heading toward a frontoparallel plane (Fig 3a; top-right inset) to illustrate how the model mechanisms work. The plot in Fig 3a addresses cells that have receptive fields centered on the left side of the display. The model MSTd cell tuned to the radial pattern and increasing speed gradient depicted in Fig 3a would maximally suppress *MT*^−^ units tuned to fast motion moving leftward (blue curve peak); cells that share an overlapping receptive field position, but have different direction (non-peak positions along the blue curve) and speed (non-blue curves) tuning would be inhibited by a lesser extent. The more dissimilar the cells’ speed and direction tuning from the combination that locally corresponds to the estimated self-motion pattern (fast motion to the left), the weaker the suppression.

**Fig 6:**
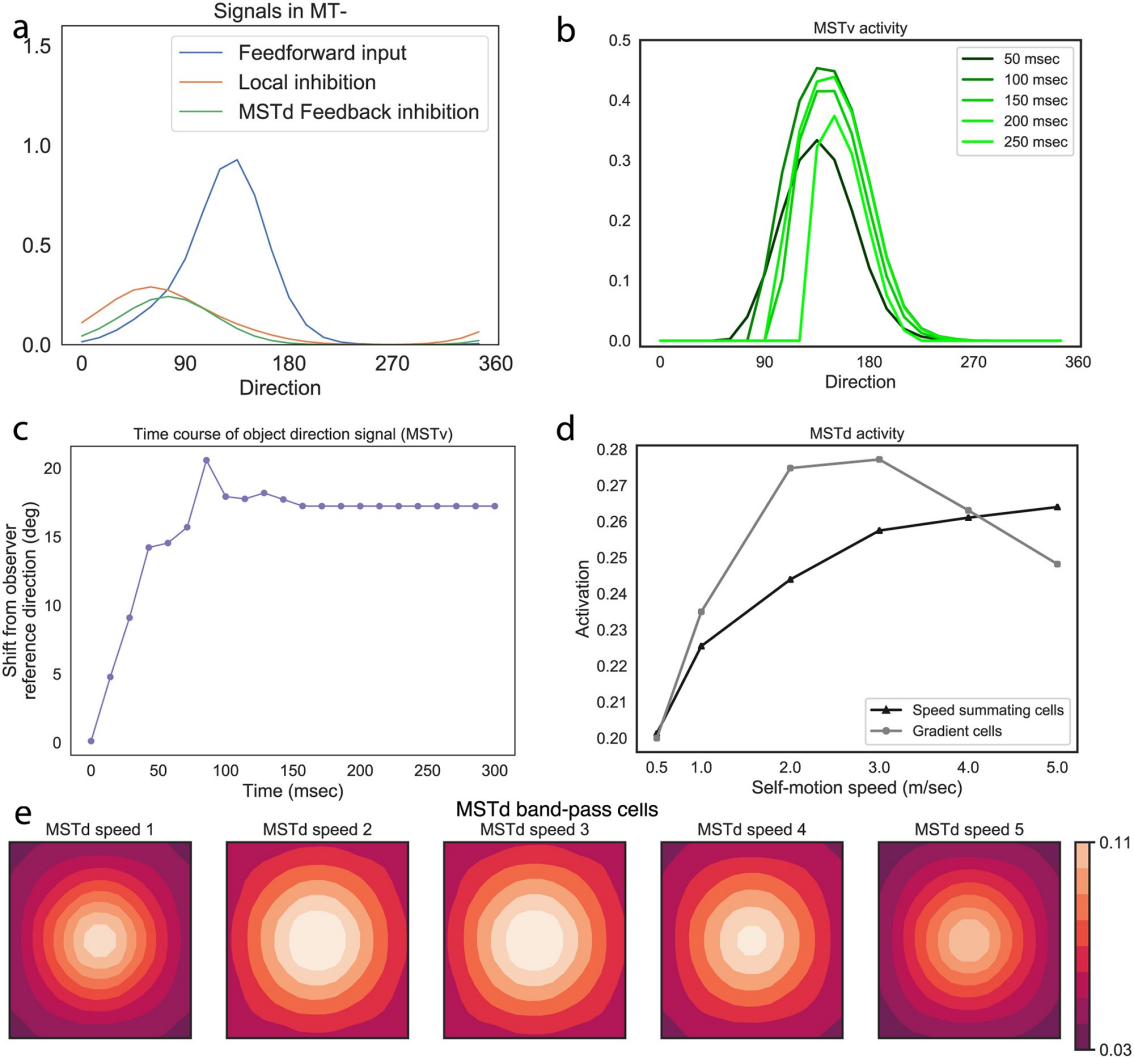
(a) Object motion signals in *MT*^−^ during fronotoparallel plane simulation. Blue is the object motion signal in *MT*^−^, green is the inhibitory feedback signal from MSTd, red is the surround (local) inhibition within *MT*^−^. y-axis is activation, x-axis is direction (0-360 deg in 15 deg increments). (b) Shift in the MSTv activity distribution over time (*MT*^−^ demonstrates a qualitatively similar evolution). (c) Time course of object motion direction represented by area MSTv units. A zero shift value indicates object motion in a observer-relative reference; positive values indicate shifts in the world-relative direction. Self-motion speed is 1 m/sec and object motion speed is 6°/s. (d) Activation of the most active gradient (gray) and speed summating (black) MSTd cells for the different self-motion speeds from the Experiment 1 frontoparallel plane stimuli. (e) MSTd band-pass cell activity that reflects an estimate of observer’s self-motion direction. The x and y axes correspond to the spatial coordinates of the optic flow. The overall activation is weak and variance in the distribution is high, indicating uncertainty in the self-motion estimate.

**Object motion signals** (*MT*^−^/*MSTv*). Fig 6a shows model inhibitory signals in a simulation of a similar frontoparallel plane scenario, but one that includes a moving object (6°/s object, self-motion speed of 1 m/s). The signals correspond to model MT^−^ feedforward surround inhibition (red curve) and suppressive feedback from MSTd (green curve). We also plot the excitatory feedforward signal that is integrated by MT^−^ units whose RFs are centered on the moving object (blue curve). The object direction represented by the excitatory blue curve is in an observer-relative reference frame. The feedback signal reflects suppression that is consistent with the direction of the estimated self-motion pattern at the local position within the visual field where the object resides, similar to Fig 3a. Together, the two inhibitory signals interact with the excitatory feedforward signal (blue) over time to shift the direction represented by the population over time, as shown in Fig 6b. In Fig 6c, we plot the direction represented across the local MSTv cell population (the next area in the model that integrates MT^−^ signals) with RFs centered on the object over time. This demonstrates an evolving shift toward the world-relative direction.

**Self-motion signals (MSTd)**. Fig 6d plots the peak activity generated by gradient (gray) and speed summating (black) cells as a function of simulated self-motion speed. Gradient cell responses ramped up as the dynamic range of images speeds broadened due to the faster self-motion speeds. Responses attenuated somewhat at higher self-motion speeds as the distribution became skewed toward faster image speeds.

Consistent with known neurophysiological properties of speed summating cells [25, 26], linear increases in speed produce a logarithmic firing rate curve in MSTd (Fig 6d). While it is not clear from the neural data whether this profile persists beyond the fastest speed tested, the firing rate of model cells plateaus. Because the amount of surround suppression from MSTd feedback depends on MSTd activation strength, this results in suppressive feedback signals that grow proportional to the slow and moderate self-motion speeds, but taper off at fast speeds. Speed summating cells therefore contribute to the increasing angular shifts produced by the model that occur when the stimulus self-motion speed increases (Fig 5b). However, the saturating activity of speed summating cells at the faster speeds prevents the model from fully compensating for the self-motion component, which becomes a greater proportion of the object’s retinal motion at faster self-motion speeds. Together with the decreasing MSTd gradient cell activity (Fig 6d), this results in shifts in the object angle that increase, but at a diminished rate, as the speed of self-motion increases (Fig 5b), consistent with the human data. This offers an intriguing possible explanation for the weak flow parsing gains at faster self motion speeds observed in the human data (≈0.3–0.4; see Fig 5a).

Unlike speed summating cells, band-pass MSTd units generate comparably weak patterns of activity across the self-motion speeds (Fig 6e; see scale of right color bar). While the centrally positioned activity peaks correctly coincide with the observer’s simulated heading toward the center of the visual display, the activity distribution across the stimulus optic flow space is broad, which indicates uncertainty about the self-motion estimate. Because MSTd units compete with one another to resolve the observer’s most likely direction of self-motion in a network that normalizes activity across the population, broader, more uncertain self-motion signals accompany weaker overall activity levels. As such, a broader MSTd activity distribution results in a weaker feedback signal, whereas a more concentrated MSTd activity distribution results in stronger feedback and may result in greater modulation of object motion signals. This means that band-pass cells only contribute a modest amount in the suppressive signal that attempts to cancel out the self-motion component from model object motion signals.

Fig 5 summarizes the simulation results across all the stimulus conditions in the form of the flow parsing gain measure (a) and the angular deviations from the observer-relative retinal direction (b). Simulations produced object direction estimates that were consistent with the human data, though there was a weak tendency to overestimate the object angle in the 2°/s condition (Fig 5b, red curve). The difference was most pronounced at moderate-to-fast self-motion speeds, when the object speed was slow compared to nearby motion vectors generated from the observer’s self-motion (e.g. Fig 4a, right column). The angular overestimate occurred because MSTd feedback, with the present selection of model parameters, overly suppressed cells tuned to speeds that are substantially slower than the nearby motion in the global motion pattern. The overall magnitude of the bias, however, in the 2°/s condition is small (mean: 4°).

The consistency in the angular deviations with the human data suggests that a self-motion signal derived from MSTd gradient, speed summating, and band-pass cells may be sufficient for capturing human object judgments in the case of the frontoparallel plane displays. Note that small offsets in angular deviation correspond to large flow parsing gain offsets, but that the flow parsing gain results also qualitatively follow the human data. The activity in each MSTd subpopulation was weak, which suggests that the single plane does not represent an effective stimulus for activating these MSTd cell types. The broadness of the activity distributions may be due to uncertainty in depth, as we will investigate in forthcoming sections.

### Simulation 2: The influence of motion parallax on accuracy of object motion perception

**Fig 7:**
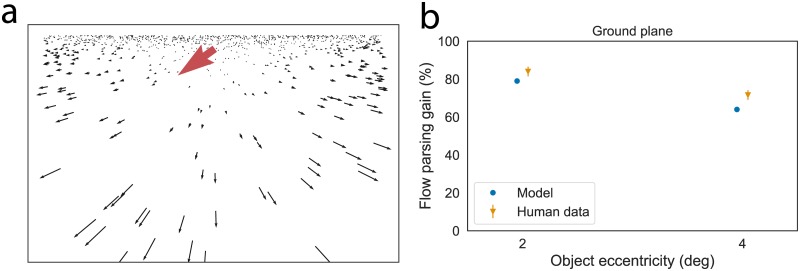
(a) Optic flow from the ground plane scenario used in Simulation 2. The red arrow shows the observer-relative motion of a small moving object. We scaled the length of the object vector 3x for visibility. (b) Model and human flow parsing gains. Error bars denote SEM.

In Simulation 2, we investigated MSTd dynamics with a stimulus that contains continuous depth variation. The optic flow corresponds to the motion parallax experienced by simulated observer translation over a ground plane (Fig 7a). The stimulus is cyclopean in the present section; we consider the addition of binocular disparity in Simulation 3.

**Fig 8:**
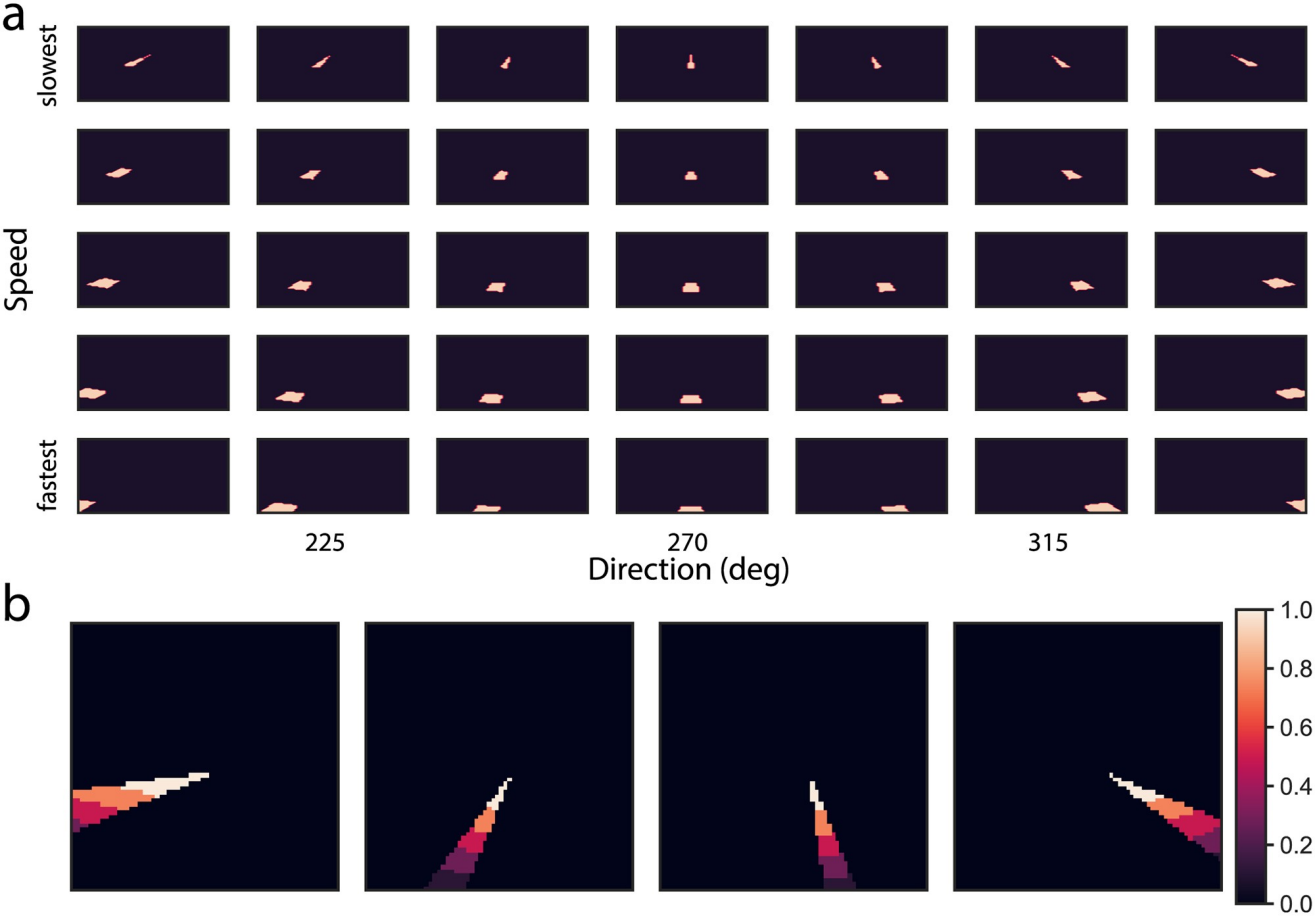
MSTd ground units. (a) Regions of a ground unit’s RF tuned to a central heading that exhibit maximal sensitivity to the indicated *MT*^+^ speeds and directions. (b) Connection weights among the different speed-tuned inputs along a subset of the directions for the slowest speed subunit. Weights shown prior to normalization.

The model configuration remained the same, except for the addition of cells that are sensitive to ground flow (‘ground units’), as has been demonstrated by physiological studies [32, 33]. As we will show, ground flow was not an effective stimulus for any of the MSTd units tuned to full-field planar radial patterns. Aside from differences in pattern sensitivity (ground vs. frontoparallel plane), the ground units integrate feedforward signals in a similar manner as model MSTd gradient cells: they not only integrate direction signals consistent with the preferred ground pattern, but the input motion signals must come from *MT*^+^ units tuned to the appropriate speed at different eccentricities. Fig 8a shows the direction and speed subunits for a single ground unit tuned to a central heading. The bright region in each subpanel shows where the *MT*^+^ input comes from within the RF. For example, the central column indicates that preferred inputs come from *MT*^+^ units tuned to downward motion (270°), and the speed preference of afferent cells increases with eccentricity (moving down the column). Consistent with full-field gradient cells (Fig 4b), non-preferred *MT*^+^ inputs to ground cells are down-weighted based on the degree of dissimilarity. Fig 8b shows the weighting for inputs tuned to the slowest speed, which appear near the preferred FOE position in the center of the RF; weights from other speed-tuned inputs decrease with eccentricity.

In addition to investigating model MSTd responses in the ground plane scenario, we used the model to explore important discrepancies in the object motion human judgments obtained by Niehorster [21] across the frontoparallel plane and ground plane experiments. In particular, self-motion component speeds of 1°/s (2° object eccentricity condition) and 2°/s (4° object eccentricity condition) for the ground plane stimuli yielded 84% and 72% mean flow parsing gains in the human data (Fig 7b), respectively. This differs markedly from the corresponding mean flow parsing gains in the frontoparallel plane scenario of 67% and 50% (Fig 5a), respectively.

#### Simulation 2: Methods

Niehorster [21, Chap. 4] has run an experiment in which static observers viewed stimuli simulating travel over a ground plane (depth range 0.56–25 m) consisting of dots while a probe dot moved linearly over this ground plane. The probe dot was placed 2° or 4° below the FOE of the flow field and moved rightward at a constant speed on the ground plane that corresponded to an initial optical speed of 2.5°/s. Two different simulated translation speeds, 5.7 m/s and 2.9 m/s (producing self-motion component speeds of 1°/s and 2°/s, respectively), were used. As before, human observers made judgments about the trajectory of the moving probe object according to the procedure described in the methods section (see Retinal Motion Nulling in General Methods).

#### Simulation 2: Model ground units

We introduced MSTd units sensitive to the optic flow generated by self-motion over a ground plane to accommodate the displays in Simulation 2. The motion pattern is proportional to the vector field

$$\begin{array}{c} \Lambda_{i,j,x,y}=\{v_{gnd,dx},v_{gnd,dy}\}=\left(\left(x-i\right)\left(y-j\right),{\left(y-j\right)}^{2}\right) \end{array}$$ (8)

in which (*i*, *j*) represents the direction of travel and Λ_*i*,*j*, *x*, *y*_ is only defined for *y* ≤ *j* (i.e. at the horizon and below). We generated motion patterns as specified by Eq 8 and extracted the directions and speeds using Eqs. S2 and S3 (see Supporting Information). We then used a procedure that is similar to that used to create MSTd gradient cell units: we generated direction templates *T*_*d*,*i*,*j*,*x*,*y*_ according to Eqs. S13 and S14, substituting Λ_*i*,*j*,*x*,*y*_ for the radial pattern *χ*_*i*,*j*,*x*,*y*_, and generated speed templates *E*_*s*,*i*,*j*,*x*,*y*_ according to Eqs. S16–S18, substituting the ground speed gradient and quintiles for *ρ*_*x*,*y*,0_ and $\rho_{s}^{4,6}$, respectively. To account for the sparsity of the ground templates (i.e. no flow defined above the horizon), we normalized by the number *MT*^+^ inputs in each ground template rather than the total number of cells with distinct RF positions across the visual field ($\hat{x}\hat{y}$) in Eq. S14. We plugged these direction and speed templates into the function $I_{i,j}^{GC}\left(\cdot \right)$ (Eq. S20), along with the *MT*^+^ activity $N_{x,y,d,s}^{+}$. The MSTd dynamics and feedback connectivity are directly analogous to those of the gradient cells.

#### Simulation 2: Simulation results

**Fig 9:**
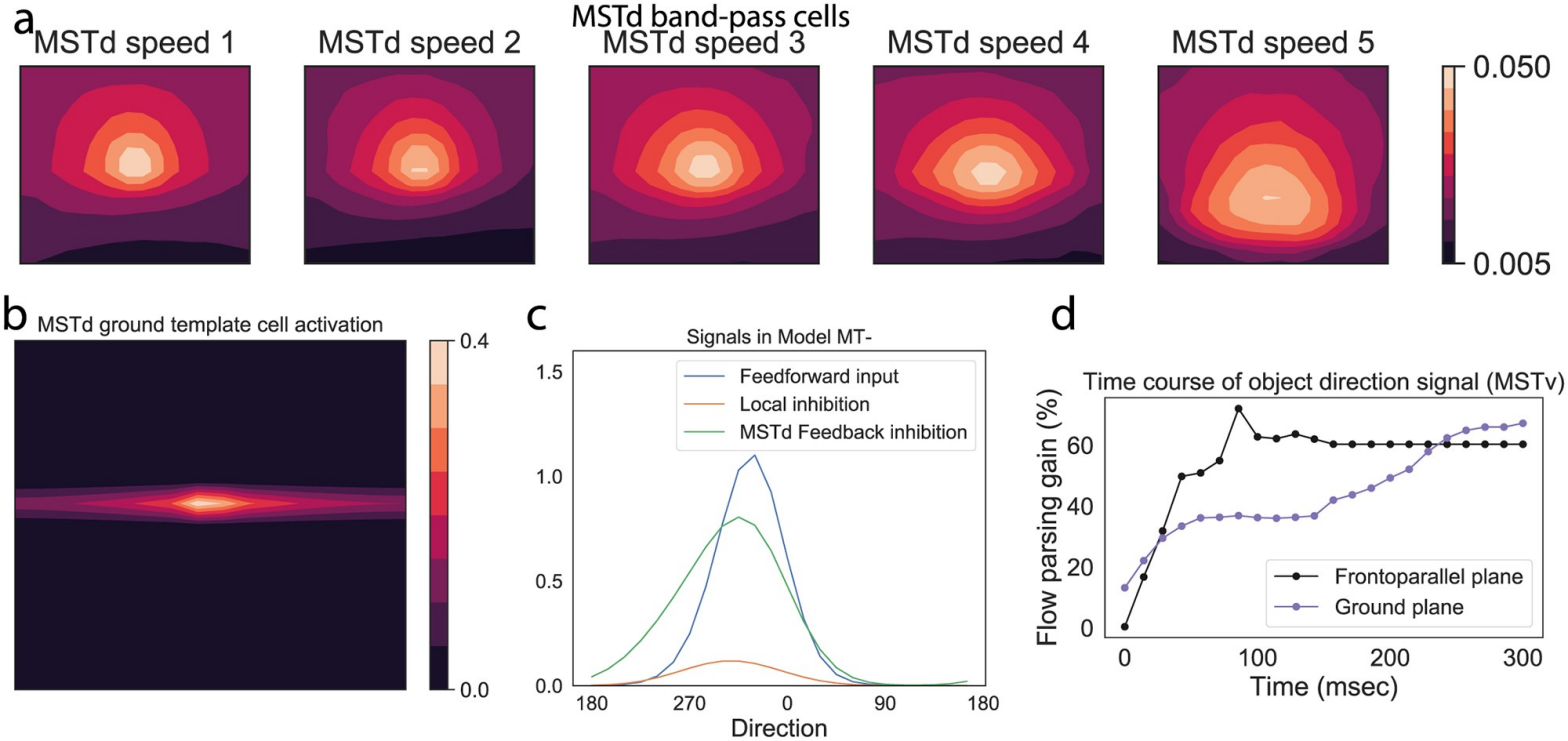
Model results for ground plane scenario (Simulation 2). (a) MSTd band-pass cell activity. (b) MSTd ground unit activity. (c) Excitatory and inhibitory signals integrated by MT units that process object motion. (d) Time course of the shift in the object direction signal represented in the model MSTv toward the world-relative direction. Black curve (frontoparallel plane) reproduced from Fig 6c. Blue curve corresponds to simulation of 4° object ground plane condition.

The optic flow in the ground plane scenario only weakly activated model MSTd band-pass, speed summating, and gradient cells. Fig 9a shows the activity for different band-pass units, which garnered the highest activity within the group. Band-pass cell activity was about half that obtained in Simulation 1 (Fig 6e) and was spatially dispersed across the subpopulation, indicating uncertainty in the self-motion estimate, particularly above the horizon.

This contrasts with the concentrated activity among ground units (Fig 9b) that signal the observer’s central heading direction, indicating higher confidence in the self-motion estimate compared to the frontoparallel plane stimuli. Because MSTd units compete with one another in a network that normalizes activity, the concentrated activity goes hand-and-hand with a stronger activity peak that was ≈33% greater than the maximum from Simulation 1. Greater peak MSTd activity resulted in stronger suppressive feedback to units in *MT*^−^ and *MSTv*. This is evident in the greater ratio between inhibitory feedback to excitatory input signal integrated by *MT*^−^ units with RFs centered on the moving object (compare red and blue curves in Figs 9c and 6a). The stronger inhibitory feedback signal induces a larger shift in the object motion signal over time (Fig 9d).

The larger MSTd activity peak in the ground plane scenario combined with the greater inhibitory feedback signal results in a flow parsing gain that is consistent with the pattern in the human data (Fig 7b). It is worth noting that the smaller model gain for the object at 4° eccentricity than the object at 2° eccentricity (Fig 7b) occurred because of the distance-dependent model function that decreases feedback weights to *MT*^−^/*MSTv* cells that have receptive fields faraway from the preferred singularity position of the maximally active MSTd cell (Eq. S28).

The close correspondence between the model flow parsing gains and the human data in both the frontoparallel plane (Fig 5a) and ground plane (Fig 7b) scenarios suggests that increased suppression through improved global pattern selectivity could explain the discrepancy in the magnitude of human flow parsing gains obtained across the stimuli used in Simulations 1 and 2. The broad and relatively weak MSTd activity in the frontoparallel plane scenario limited the extent to which self-motion signals could transform object motion signals from retinal to world-relative reference frames through the surround suppression mechanism. Increased surround suppression in the ground plane scenario due to the greater MSTd activity and increased pattern selectivity allowed the model to factor out a greater proportion of the observer’s self-motion, thereby improving the accuracy of object motion signals.

### Simulation 3: The influence of binocular disparity on accuracy of object motion perception

**Fig 10:**
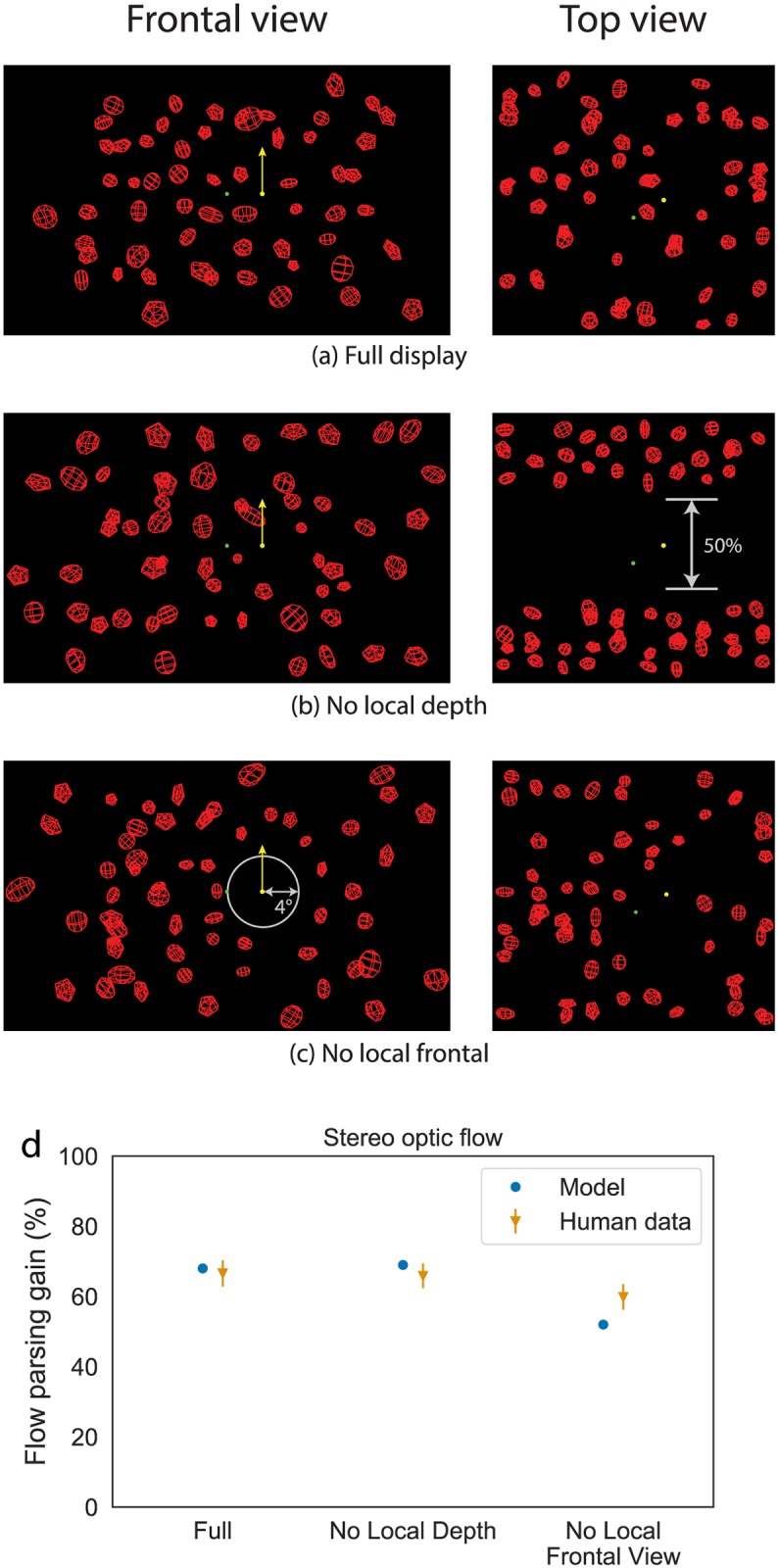
(a) Full, (b) No Local Depth, and (c) No Local Frontal View conditions (Simulation 3). Reproduced from Niehorster & Li [2]. (d) The flow parsing gain achieved for these conditions by the model and the human observers. Error bars denote SEM.

Simulation 3 focuses on how model MSTd processes optic flow that accompanies motion parallax with binocular disparity. We simulated stereo visual displays from Niehorster & Li [2] consisting of wire-frame cubes arranged in a 3D volume (Fig 10a–10c). We focused on three particular conditions to probe the contributions of local (‘No Local Frontal View’ condition) and global (‘Full’ condition) surround suppression signals as well as the role of local depth (‘No Local Depth’ condition). The Full condition includes full-field stereo optic flow and should fully recruit the model mechanisms. The No Local Depth condition removes a cross-section of cubes at depths nearby the moving object and should probe the contributions of local depth signals in the model. The No Local Frontal View condition removes a local 4° volume surround the moving object and should inactivate local feedforward inhibition.

We also used the model to investigate an apparent conflict in the human data with respect to whether the accuracy of self-motion perception depends on the speed of self-motion. While the human frontoparallel plane data from Niehorster [21] used in Simulation 1 (Fig 5a) show a dependence of self-motion speed on flow parsing gain, the data on the stereo wire-frame scenario from Niehorster & Li [2] showed no such decline. We therefore furthermore simulated the stereo conditions of Niehorster & Li [2] that vary the speed of self-motion to compare model dynamics with those obtained in the frontoparallel plane scenario (Simulation 1).

#### Simulation 3: Experimental data

Niehorster & Li [2] had static observers view stimuli simulating travel at 0.30 m/s through a cloud of wire-frame objects (depth range 0.69–1.03 m) while a probe dot moved linearly through this cloud. The scene was rendered in stereo and four display conditions were presented to observers in which the wire-frame objects were distributed differently in the cloud. Three of these conditions are used for the current simulations: 1) a *Full* condition in which the wire-frame objects were distributed throughout the entire depth range of the cloud; 2) a *No Local Depth* condition in which no objects were placed in the central 50% of the depth range of the cloud; and 3) a *No Local Frontal View* condition in which no objects were placed within 4° from the probe in the frontal view of the display. The probe dot was visible during the last 200 ms of each 1 s trial and was placed 4° to the left or right of the FOE of the display, at the center depth of the cloud. The probe moved linearly upward in the cloud at a speed that corresponded to a vertical optical speed of 2°/s at the midpoint of the probe’s trajectory, at which moment the horizontal optical speed component of the probe due to simulated self-motion was also 2°/s. As before, human observers made judgments about the trajectory of the moving probe object according to the procedure described in the methods section (see Retinal Motion Nulling in General Methods).

In their Experiment 2, Niehorster & Li [2] furthermore had static observers perform the same task using the Full display condition while varying simulated self-motion speed. Translation speeds of 0.24 m/s, 0.39 m/s, 0.54 m/s, and 0.69 m/s were used which corresponded to a horizontal optical speed component of the probe due to simulated self-motion of 1.6°/s, 2.6°/s, 3.6°/s, and 4.6°/s, respectively.

We used the motion of the vertices of the wireframe objects as input to the model.

#### Simulation 3: Model disparity tuning

We made two overarching changes to augment the model with disparity tuning in areas MT and MST. First, we designed separable disparity filters and multiplicatively combined them with those for direction and speed to produce units throughout the model that are jointly tuned along all three dimensions. This is consistent with neural data showing that disparity tuning in MT [34] and in many cases in MSTd [30] is largely independent of speed and direction. Second, we introduced a disparity-dependent gain (Fig 2d) in MT and MSTd signals to account for the tendency for neural responses to be greater for an optic flow stimulus viewed binocularly than when viewed monocularly in otherwise similar conditions [20]. The gain decreased with depth, consistent with the weak tendency for disparity sensitivity to become coarser with depth [35]. Supporting Information S1 details the specific modifications that were made.

#### Simulation 3: Results

**Fig 11:**
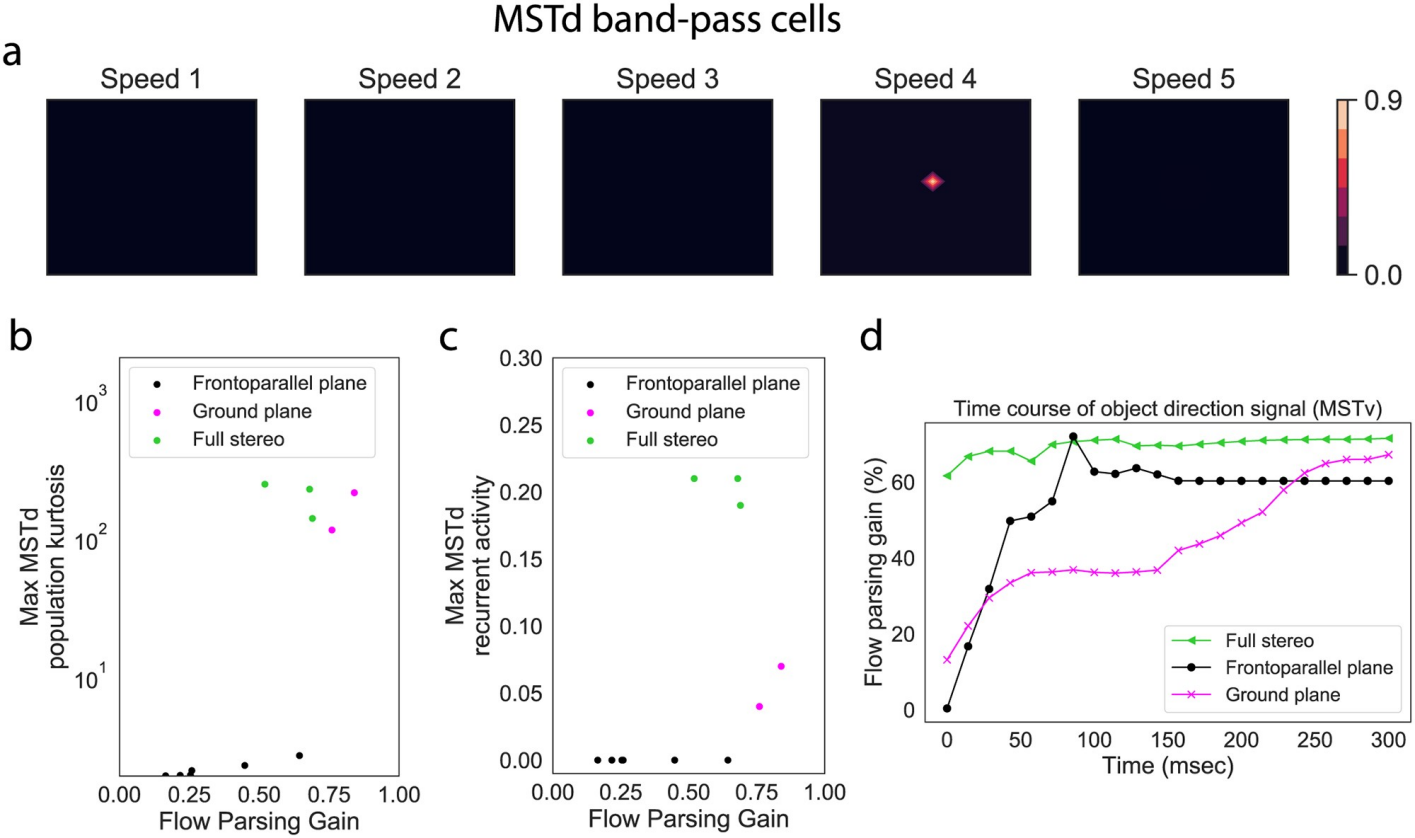
(a) MSTd band-pass cell activity for the Full Stereo condition. (b) Kurtosis of the most active MSTd subpopulation response, which measures the peakedness of distribution, in each simulation. (c) Strength of the recurrent signal generated by the most active MSTd subpopulation in each simulation (Eq. S21). A zero value in this panel indicates that the MSTd signal did reach the appropriate threshold to engage the recurrent mechanism. (d) Time course of the object direction signal in model MSTv. Black and blue curves reproduced from Fig 9d.

Fig 11a plots the activity of model MSTd band-pass cells tuned to the disparity that matches that of the object in the Full condition. The activity distribution is highly peaked, indicating confidence in the self-motion estimate. Fig 11b quantifies this using kurtosis, defined as the normalized fourth moment of each population response distribution. The plot demonstrates increased kurtosis in each subpopulation response, compared to all responses in the frontoparallel plane scenarios and most ground plane scenarios (Simulations 1 and 2). This means that MSTd units showed increased selectivity to the global motion pattern that corresponds to the observer’s self-motion. Due to the normalizing property of the MSTd competitive network, this means that the unit that signals the self-motion estimate sends a stronger signal than in the frontoparallel and ground plane simulations. Crucially, MSTd units require a strong enough input to engage the recurrent mechanism (see threshold in Eq. S21); otherwise the competition would pathologically enhance weak, noisy, and unreliable motion signals. The disparity-dependent gain in MT signals allowed the network to more fully recruit the recurrent mechanism than in the frontoparallel and ground plane scenarios (Fig 11c).

Fig 11d shows the shift in the object motion signal toward a world-relative reference frame in the Full condition. The stronger suppressive feedback signal induced by the enhanced MSTd self-motion estimate resulted in greater and earlier modulation in the object direction than in the frontoparallel and ground plane scenarios. The modulation plateaued after ≈150 msec due to saturation in the maximally active MSTd unit: it could not generate a much stronger suppressive feedback signal (see Fig 11a; panel 4).

**Fig 12:**
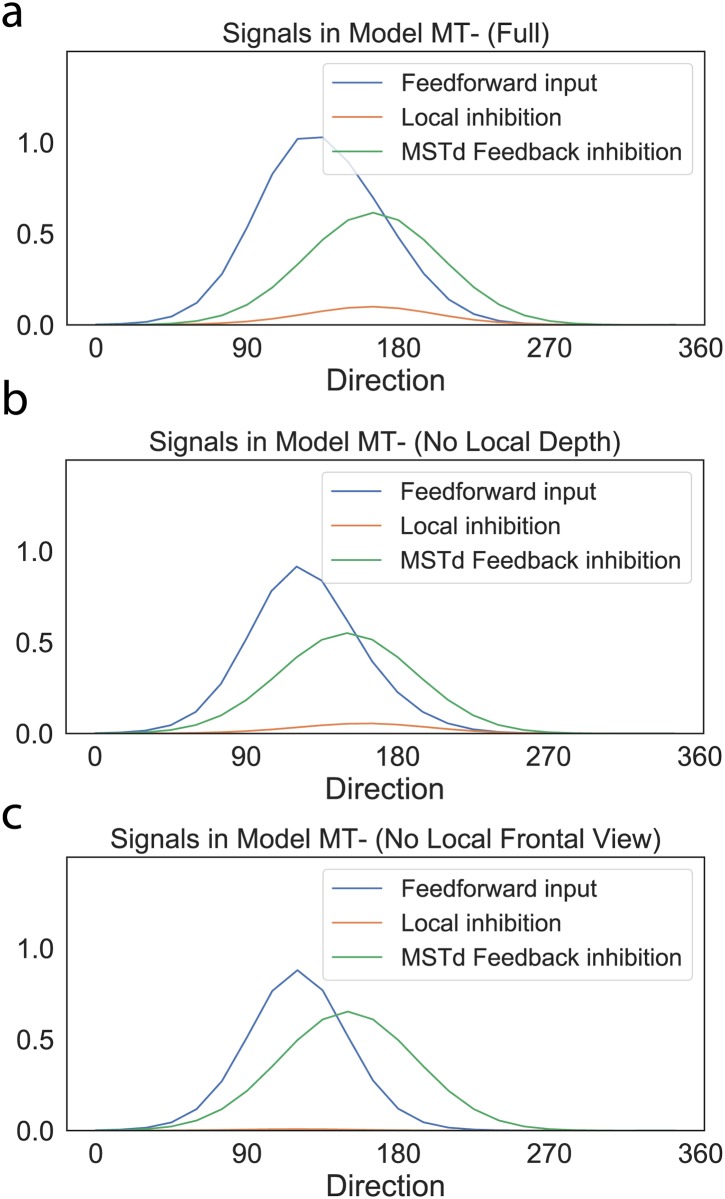
Object motion signals in MT in the (a) Full Stereo, (b) No Local Depth, and (c) No Local Frontal View conditions. Blue is the object motion signal in *MT*^−^, green is the inhibitory feedback signal from MSTd, red is the surround (local) inhibition within MT. y-axis is activation, x-axis is direction (0–360° in 15° increments). Local inhibition refers to the inhibitory signal due to feedforward lateral inhibition from units tuned to similar directions, speeds, and disparities in MT Layer 4/6.

Fig 12 plots the feedback signal, along with the other excitatory and inhibitory signals integrated by *MT*^−^ units that have the moving object in their receptive fields in the Full, No Local Depth, and No Local Frontal View conditions. Given the similarity in the global flow across the conditions, the suppressive feedback signal from MSTd remains largely unchanged. However, removing only the fiducial points at nearby depths from the object diminishes, but does not eliminate, surround disparity signals, which yields weak local inhibition (Fig 12b). Locally removing elements throughout the volume abolishes local inhibitory signals (Fig 12c). Across the three conditions, the direction represented by model object motion signals yielded flow parsing gains that correspond to human judgments (Fig 10d).

**Fig 13:**
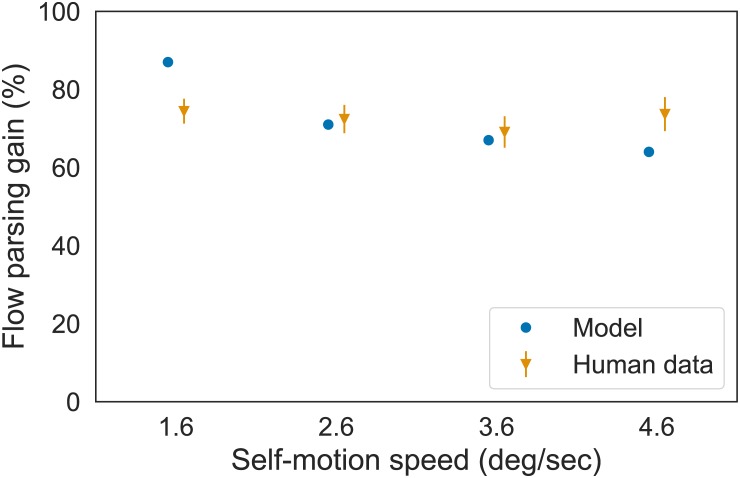
The flow parsing gain achieved by the model and the human observers for different self-motion speeds with stereo optic flow (Simulation 3). Error bars denote SEM.

We simulated four additional stereo conditions of Niehorster & Li [2] to focus on the issue of whether object motion perception depends on the speed of self-motion (Fig 13). As the speed of self-motion increases, so does its relative contribution to the object’s retinal motion potentially making it more challenging to factor out the appropriate amount of the self-motion component. In the frontoparallel plane scenario, MSTd activity was relatively weak (Fig 6d and 6e), preventing the model from factoring out enough of the self-motion component at faster speeds. In the stereo scenario, however, the model demonstrates a weak dependence on the self-motion speed (Fig 13). This occurred because the stronger motion signals in the stereo simulation engaged the recurrent mechanism, which enhanced the MSTd self-motion estimate and allowed the model to compensate for the larger self-motion component proportion over a broad range of self-motion speeds.

## Discussion

Neurophysiological studies have identified subpopulations of neurons in primate MSTd tuned to characteristic patterns of direction, speed, and disparity. Using a neural model that simulates key functional properties of these neurons, we demonstrated that these MSTd neurons, along with local inhibitory signals in MT, may explain human object motion perception during simulated self-motion through scenes with different monocular and binocular depth cues. The frontoparallel plane scenario that created ambiguity about depth (no depth variation and plane at an indeterminate depth) elicited weak-to-moderate MSTd activity across the different subpopulations. This resulted in weak modulation of the object motion signal, especially at faster self-motion speeds, consistent with human judgments about object direction. Simulated self-motion over a ground plane (continuous depth variation) resulted in more vigorous and selective responses in MSTd. The most active MSTd units sent stronger feedback signals that better suppressed the self-motion component and resulted in more accurate scene-relative object motion signals in MT and MSTv. Finally, we examined how disparity dependent changes in MT signal gain influence object motion responses within our model of MT/MST. We found that increases in MT signal gain not only increased the strength of MSTd responses, but enhanced their selectivity (Fig 11b). This was because the elevated disparity dependent gain more effectively recruited recurrent feedback mechanisms within MSTd (Fig 11c). The increased selectivity in turn led to higher model flow parsing gains than in the other two simulations.

The correspondence between the direction represented in model object motion signals and human judgments (Figs 10d and 13) suggests that improved global flow estimation within a *MT*^−^MSTd neural circuit similar to that in the proposed model could serve as a mechanism for perceiving world-relative object motion perception during self-motion. In humans, the areas may correspond to the MT complex and area V6, respectively [36]. Precise depth information (e.g. through binocular disparity) may facilitate this process through enhanced identification of the global motion pattern due to the observer’s self-motion in MSTd. That is, the presence of speed gradients, binocular disparity, and other sources of reliable depth information may do more than simply increase the gain of self-motion signals—it suppresses the activity of MSTd units tuned to combinations of motion parallax and disparities that are unlikely to correspond to the observer’s self-motion. This can be appreciated in Fig 11b, which summarizes the kurtosis (peakedness) of the MSTd population, a measure of model pattern selectivity, across our simulations.

### Does object motion perception during self-motion depend on the speed of self-motion?

Our simulations reconcile an apparent discrepancy between the data from Niehorster [21] showing that flow parsing gain decreases as the speed of self-motion increases (Fig 5a) and those from Niehorster & Li [2] that exhibit no such relationship (Fig 10d). As the observer’s speed of self-motion increases, so does the proportion of self-motion component in the object’s retinal motion. This increases the demands on mechanisms that factor out the self-motion component to recover world-relative object motion. Considering how model MSTd responses saturated and even declined at faster self-motion speeds in the frontoparallel plane scenario (Niehorster [21]; Fig 6d), the feedback mediated mechanism proposed here cannot send a strong enough signal up with the amount of suppression that is required to support accurate object motion signals. The disparity-dependent gain in the stereo scenario (data from Niehorster & Li [2]; Fig 12a) allowed the model to effectively suppress the self-motion component over a larger range of self-motion speeds due to the recurrent dynamics. The normalizing property of the recurrent MSTd network leads to the prediction that there may be a limiting factor or bottleneck in the recovery of world-relative object motion at even faster self-motion speeds. That is, the flow parsing gain may eventually decline at faster self-motion speeds than those examined here.

### Global pattern estimation and heading

Our results are consistent with the hypothesis that depth information allows the visual system to better estimate the retinal pattern of motion during self-motion. That is, different environments and viewing conditions may enable neurons with RFs positioned in different locations across the visual field to generate direction, speed, and disparity signals that better capture the observer’s world-relative movement. This does not necessarily imply improved heading estimation or sensitivity to the singularity position (e.g. FOE) within the global optic flow field.

Indeed, heading estimation is well-known to be accurate in sparse, improvised environments [37]. In the model mechanisms proposed here, many MSTd neurons may exhibit sensitivity to the observer’s heading, but far fewer may integrate the appropriate direction, speed, and disparity across the visual field to accurately signal the global pattern that arises due to the observer’s self-motion. The specificity in tuning to optic flow along these dimensions is important because moving objects may occupy any region of the visual field and yet the visual system must still factor out the appropriate amount of self-motion component.

The proposed model focuses on patterns of self-motion induced by observer translation; it does not directly address optic flow that contains a combination of translation and rotation that may arise during eye and head movements or movement along a curved path. It is presently unclear the extent to which humans recover world-relative object motion when the global pattern contains both translation and rotation, considering that human object motion perception has only been studied in a limited range of simplistic conditions [38]. However, assuming humans are capable of world-relative object motion perception in the presence of both translation and rotation, in principle, the visual system could rely on a solution that solely depends on non-visual (e.g. vestibular) signals, one that that solely depends on vision, or one that depends on a combination thereof.

Considering that non-visual information improves the accuracy of world-relative object motion perception in humans [10, 12, 39] and MSTd neurons exhibit systematic vestibular tuning [40], multisensory integration may play an important role in enhancing the specificity of the estimated self-motion pattern, especially when it contains rotation [41]. Indeed, congruence between visual-vestibular signals appears to improve selectivity, perhaps somewhat akin to the effects of increased MT signal gain in the model [20]. Multisensory integration may also facilitate the recovery of world-relative object motion when the visual signal is weak or noisy (e.g. due to impoverished depth information or in low light). Additional studies are needed to assess the contributions of multisensory tuning on the object motion responses.

On the other hand, physiological evidence demonstrates that MSTd neurons are capable of estimating the contributions of rotation using visual signals alone [42]. This suggests that non-visual signals may not be necessary in a process that recovers world-relative object motion when the global motion pattern contains rotation. Computational models of vision have proposed two broad classes of solutions for addressing the presence of rotation, which have different implications for recovering object motion signals: removing rotation before motion signals reach MSTd [43] and estimating the rotation within MSTd [44–46]. The former approach has proposed that rotation is removed within MT through a local subtractive process, perhaps implemented with *MT*^−^ inhibitory surrounds. In the context of the model proposed here, subtraction in MT would remove the rotational component and MSTd feedback could suppress the remaining global translational component. By the alternative account, rotation could be estimated within MSTd using cells sensitive to global laminar motion. Together with cells that respond to the translational component, these cells could send feedback to *MT*^−^ and MSTv to suppress the retinal motion due to the observer’s self-motion. This is an important topic that warrants further investigation.

### Distinct pathways for self-motion and object motion

Evidence of two anatomically segregated subpopulations in MT—cells with reinforcing surrounds (*MT*^+^) and those with suppressive (*MT*^−^) surrounds [17, 18]—guide the structure of proposed model. It has been suggested that these cell types perform complementary functions: *MT*^+^ cells may specialize in processing uniform ‘wide-field’ motion, whereas *MT*^−^ cells may specialize in processing local motion contrast [14, 15, 28, 47]. This functional specialization may contribute to self-motion and object motion sensitivity in areas further along in the visual system. Indeed, Born and colleagues have provided evidence that among *MT*^+^ and *MT*^−^ cells, a higher proportion of *MT*^+^ cells project to MSTd and a higher proportion of *MT*^−^ cells project to MSTv [48]. Yu and colleagues have recently supported this finding by demonstrating that electrical microstimulation of *MT*^+^ cells influenced monkey heading judgments to a greater extent than *MT*^−^ cells [22]. The present model implements these apparent *MT*^+^–MSTd and *MT*^−^–MSTv pathways in a ‘strong’ sense in that we do not include overlapping projections (i.e., *MT*^−^–MSTd and *MT*^+^–MSTv). This omission may accentuate MSTd/MSTv activation, potentially contributing to instances wherein the model overestimates the object angle (e.g. Figs 5 and 13).

Mixed projections may facilitate the extent to which MSTd/MSTv could differentiate between self-motion and object motion. For example, physiological [49] and modeling [13, 43, 50] studies have shown that large moving objects influence heading estimates produced by MSTd neurons and it is possible to decode self-motion and object motion using visual signals alone [49]. Band-pass cells in the present model with their differing speed selectivities may facilitate this process in scenarios wherein the observer’s self-motion activates one group of MSTd cells (e.g. Fig 11a) and the moving object activates a second group. Because moving objects often occupy a smaller area of the visual field than the flow created by observer’s self-motion relative to the stationary environment, we would expect the most active cells to represent the observer’s self-motion and the second most active cells to represent the object motion. Interactions between these simultaneously active cells may help resolve uncertainty about self-motion and object motion. Considering that the moving objects in the present study were too small to influence MSTd activation, this idea remains to be tested.

### Pattern selectivity in MSTd

Despite the classical emphasis on radial or laminar full-field motion sensitivity of MSTd neurons, tuning does not appear to be uniform throughout the RF [24, 51]. Individual neurons appear to integrate a milieu of excitatory and inhibitory direction inputs that are not compatible with a single coherent global pattern. Therefore, the pattern selectivity of MSTd neurons may not fall into distinct full-field expansion, ground, and other categories; neurons may instead respond to many global motion patterns with varying effectiveness. For instance, through the diversity of subunits, the same neurons may signal the global motion pattern in different scenarios (e.g. frontoparallel and ground plane scenarios). This would explain how human object motion perception remains reliable and accurate in a wide range of environments and viewing conditions. The proposed model likely underestimates the extent to which MSTd units carry out multiple functions. Future models should explore how the same units may be flexibly recruited to estimate the global motion pattern in diverse circumstances without increasing the number of distinct patterns to which cells are tuned. The present work demonstrates that in principle cells with well-characterized properties organized in distinct populations can account for human object motion perception during self-motion. It remains to be seen whether a population of cells with increased functional overlap and redundancy could exhibit similar behavior.

## Supporting information

S1 Text Mathematical specification of the neural model of MT and MST. (PDF)
