# Supplementary material for: A model of how depth facilitates scene-relative object motion perception
Source: PLoS Comput Biol. 2019 Nov 14;15(11):e1007397. doi: 10.1371/journal.pcbi.1007397 (PMC6879150; doi:10.1371/journal.pcbi.1007397)
Supplement: S1 Text — (PDF) [file pcbi.1007397.s001.pdf]

Supporting Information: Specification of neural model of MT and MST

This appendix outlines the mathematical specification of the neural model of MT and MST simulated in the present study. The model extends the Competitive Dynamics Model and builds on the ViSTARS model [1, 2] to encompass object motion during self-motion, motion parallax, and disparity [3, 4]. The sections that follow present the baseline model used in Simulation 1. Modifications for Simulation 2 are described in the main text and those for Simulation 3 are described in a section at the end of the appendix.

Notation

When defining functions, we use the notation $F(x_1, \ldots, x_n; p_1, \ldots, p_m)$, where F is the function name, $x_1, \ldots, x_n$ are the independent variables, and $p_1, \ldots, p_m$ are parameters. In expressions and equations, we use the notation $F(x_1, \ldots, x_n)^a$, where F is the name, $x_1, \ldots, x_n$ are the independent variables, and a refers to the relevant model area, if applicable. Specific parameter values used in Gaussian or von Mises tuning curves appear in Table 2, values that relate to neural dynamics appear in Table 3.

We use d to index direction preference (e.g. $d = 1, \ldots, \hat{d}, \hat{d} = 24$). Parameters that appear with a hat refer to ordinal count (e.g. $\hat{d}$ refers to the 24 MT tuning directions between 0° and 360°). The symbol θ_d refers to the specific angles to which MT units are tuned. We use s to index speed tuning preference and $\rho_s^{4,6}$ to refer to specific speeds to which units are tuned, where the superscript $4, 6$ in this case indicates that the speeds correspond to model MT Layer 4,6 (input layer). We index horizontal and vertical position in retinal coordinates by (x, y).

Considering that the input comes as a sequence of digital images, we assumed for convenience a 1-to-1 mapping between pixels in each image and the regular grid of units in retinal coordinates, meaning (x, y) also indexes the pixels along each linear dimension of an input frame. MSTd cells sample space more coarsely and we use (i, j) to index their preferred singularity position.

We assume that the model input arrives as discrete snapshots (i.e. frames of video). The model operates in continuous time t so we relate frame number $f = 1, 2, ..., n$ and t using the expression $f = \lceil t \rceil$, where $\lceil \cdot \rceil$ indicates the ceiling operation.

MT Layer 4,6

The response of units in the input layers of MT is obtained by filtering the input motion signal $\vec{v}_{x,y,f}$ with tuning curves that define their joint sensitivity to direction and speed. The MT direction tuning followed a von Mises distribution:

$$V(a; \mu, \kappa) = \frac{e^{\kappa(a-\mu)}}{e^{\kappa}} \quad (1)$$

that we evaluated at $V\left(\Theta_{x,y,f}; \theta_d, b^{4,6}\right)$ for all x, y, and f. We extract the angle of the motion vector at each position $(\Theta_{x,y,f})$ via the two argument form of the arctangent function.

$$\Theta_{x,y,f} = \text{arctan2}(v_{dy,f}, v_{dx,f}) \quad (2)$$

Eq 1 matches this angle with the preferred direction θ_d of each unit, where the parameter $b^{4,6}$ controls the direction tuning width. We normalized Eq 1 so that it outputs a maximum of 1 when the peak tuning of the unit matches the input.

MT L4,6 units process the input signal for speed by computing

$$\rho_{x,y,f} = \sqrt{v_{dx,f}^2 + v_{dy,f}^2} \tag{3}$$

and evaluating the following Gaussian tuning curve at $G\left(\rho_{x,y,f}; \rho_s^{4,6}, \sigma_s^{4,6}\right)$ for all x, y, and f:

$$G(a; \mu, \sigma) = \frac{1}{\sqrt{2\pi\sigma}} e^{-\left(\frac{a-\mu}{\sqrt{2}\sigma}\right)^2} \tag{4}$$

where $\sigma_s^{4,6}$ controls the width of the tuning curve across speeds and $\rho_s^{4,6}$ define the speeds that yield the maximal sensitivity in the MT population. As indicated in Table 1, we created units tuned to five speeds ($s = 1, 2 \ldots, \hat{s}; \hat{s} = 5$) and defined the speeds that elicit the maximal filter response according to speed distribution quintiles present in the first frame of each input ($\vec{\rho_1}$).

The speed tuning curves increased in tuning width, proportional to speed s as follows:

$$\sigma_s^{4,6} = \sigma_{spd}^{4,6}\left(1 + e^{s\sigma_{ds}^{4,6}}\right) \tag{5}$$

We computed the MT L4,6 activation $M_{x,y,d,s,f}^{4,6}$ by multiplicatively combining the input direction and speed tuning curve outputs:

$$M_{x,y,d,s,f}^{4,6} = \left[V\left(\Theta_{x,y,f}; \theta_d, b^{4,6}\right)\right]\left[G\left(\rho_{x,y,f}; \rho_s^{4,6}, \sigma_s^{4,6}\right)\right] \tag{6}$$

MT Layer 2-3 (MT^+)

MT^+ units perform a spatial integration of MT L4,6 activity $M_{x,y,d,s,f}^{4,6}$ throughout the receptive field using a two-dimensional Gaussian (2D) filter:

$$G(x, y; x_0, y_0, \sigma_x, \sigma_y) = \frac{1}{2\pi\sigma_x\sigma_y} e^{-\left(\left(\frac{x-x_0}{\sqrt{2}\sigma_x}\right)^2 + \left(\frac{y-y_0}{\sqrt{2}\sigma_y}\right)^2\right)^2} \tag{7}$$

The following 2D convolution function specifies how this integration occurs:

$$C(w_{x,y}; \mu_x, \mu_y, \sigma_{x,y}, g) = g \sum_n \sum_m G(n, m, \mu_x, \mu_y, \sigma_{x,y}, \sigma_{x,y})\, w_{x-n,y-m}, \tag{8}$$

where $w_{x-n,y-m}$ defines the input cell activity (e.g. $M^{4,6} - -MT^+$) and g represents the gain of the convolution pooling operation.

We evaluate Eq. 8 at $C_{x,y,d,s,f}^+ = C\left(M_{x,y,d,s,f}^{4,6}; x, y, \sigma^{MT^+,cent}, 1\right)$ to obtain the MT^+ input.

We defined the MT^+ activity $M_{x,y,d,s}^+$ according to the leaky integrator equation

$$\frac{dM_{x,y,d,s}^+}{dt} = -M_{x,y,d,s}^+ + \left(1 - M_{x,y,d,s}^+\right) C_{x,y,d,s,\lceil t \rceil}^+ \tag{9}$$

Among models that explain MSTd responses based on their feedforward input from MT, those that include a nonlinearity that compresses the MT signals perform

best [5]. The compressive nonlinearity could be explained by synaptic depression, the tendency for the same inputs to lose their efficacy over time. We modeled MT^+ synaptic depression $Y^+_{x,y,d,s}$ as follows:

$$\frac{1}{\tau^+}\frac{dY^+_{x,y,d,s}}{dt} = 1 - Y^+_{x,y,d,s}(1 + \kappa^+ M^+_{x,y,d,s}) \quad (10)$$

$$N^+_{x,y,d,s} = Y^+_{x,y,d,s} M^+_{x,y,d,s} \quad (11)$$

where $N^+_{x,y,d,s}$ denotes the output signal to $MSTd$ from MT^+ units, τ^+ is the synaptic time constant, and κ^+ represents the rate at which the efficacy of the input signal $M^+_{x,y,d,s}$ declines over time [6].

MSTd

Visual self-motion pattern matching $(MT^+ - MSTd)$

Model MSTd cells estimate the pattern of motion parallax that corresponds to the observer's self-motion. For the present simulations, we implemented radial pattern selectivity (Eq. 1). We created direction templates that select MT^+ signals when they appear in a location that is locally consistent with the preferred global radial pattern. For example, the rightward direction template pools the responses of MT^+ cells tuned to rightward motion when their receptive fields coincide with the right side of the visual field. The following equations define these templates $T_{d,i,j,x,y}$ that integrate MT^+ cells tuned to direction d, normalized by the total number of pooled cells $(\hat{x}\hat{y})$ (Eq. 14):

$$\chi_{i,j,x,y} = \text{arctan2}(y - j, x - i) \quad (12)$$

$$\tilde{T}_{d,i,j,x,y} = \begin{cases} 1 & \frac{2\pi(d-1-\hat{d})}{\hat{d}} < \chi_{i,j,x,y} < \frac{2\pi(d-\hat{d})}{\hat{d}} \\ 0 & \text{otherwise} \end{cases} \quad (13)$$

$$T_{d,i,j,x,y} = \frac{1}{\hat{x}\hat{y}}\tilde{T}_{d,i,j,x,y} \quad (14)$$

The following equation matches the direction templates $(T_{d,i,j,x,y})$ with the output signals from MT^+ $(N^+_{x,y,d,s})$ to resolve the direction component of the feedforward input $(R_{i,j,s})$ to the MSTd unit that prefers a singularity positioned at (i,j):

$$R_{i,j,s} = \sum_x \sum_y \sum_d e^{-b^{MSTd}((x-i)^2+(y-j)^2)} T_{d,i,j,x,y} N^+_{x,y,d,s}. \quad (15)$$

In Eq. 15, the exponential function makes MSTd units more sensitive to motion nearby the preferred singularity position and the parameter b^{MSTd} modulates how the sensitivity decreases with distance.

Speed tuning $(MT^+ - MSTd)$

Model MSTd contains units tuned for three distinct speed profiles: speed summating cells, band-pass cells, and gradient cells. The former two cells obtain their speed sensitivity by evaluating the MT^+ signal $N^+_{x,y,d,s}$ with the previously described functions Eqs. 2 and 3, respectively. Gradient cells not only match MT^+ signals that are locally consistent with the preferred radial template direction, but the activity must come from MT^+ units tuned to the appropriate speed at different eccentricities

(i.e. input from cells tuned to faster speeds at greater eccentricities). We impose the first condition by matching MT^+ signals $N^+_{x,y,d,s}$ with the direction templates $T_{d,i,j,x,y}$ (Eq. 14), and the second condition via the following annular speed templates ($E_{s,i,j,x,y}$):

$$\tilde{E}_{s,i,j,x,y} = \begin{cases} 1 & \rho^{4,6}_{s-1} \leq \rho_{x,y,0} < \rho^{4,6}_{s} \\ 0 & \text{otherwise} \end{cases} \tag{16}$$

$$\check{E}_{s,i,j,x,y} = \frac{1}{\hat{x}\hat{y}} \tilde{E}_{s,i,j,x,y} \tag{17}$$

In Eq. 16, $\rho^{4,6}$ refers to the set of speeds to which MT units that elicit the maximal responses. As described above, we configured MT in our simulations with tuning to five different speeds ($\hat{s} = 5$), and defined specific values according to the quintiles present in the first input frame $\rho_{x,y,0}$. Considering that self-motion in the present study is always forward translation, $\check{E}_{s,i,j,x,y}$ appears as five concentric, disjoint annuli centered on the singularity (i, j). To mitigate aliasing effects owing to the steep discontinuities between successive annuli, the gradient cell speed templates take a weighted sum of units tuned to similar speeds:

$$E_{s,i,j,x,y} = D(\check{E}_{s,i,j,x,y}; s, \sigma^{grad}_{spd}) \tag{18}$$

where $D(\cdot)$ refers to the 1D convolution

$$D(w_{x_0}; \mu, \sigma) = \sum_n G(n - x_0; \mu, \sigma) w_{x_0} \tag{19}$$

along the speed dimension, where σ^{grad}_{spd} represents the degree of pooling between MT cells tuned to similar speeds.

Network dynamics

The input signal to MSTd units depends on the unit's preferred motion parallax pattern. For gradient cells, the input signal is defined according to the following function $I^{GC}_{i,j}(\cdot)$:

$$I^{GC}_{i,j}(w_{x,y,s}) = \sum_x \sum_y \sum_s \sum_d T_{d,i,j,x,y} E_{s,i,j,x,y} w_{x,y,d,s} \tag{20}$$

For band-pass and speed summating cells, we composed the functions that define the direction component of the radial template match ($R_{i,j,s}$, Eq. 15) and the appropriate speed profile ($U(\cdot)$ for summating cells (Eq. 2), and $B_s(\cdot)$ for band-pass cells (Eq. 3)). For example, we generated the input to band-pass cells by evaluating $I^{BP}_{i,j,s} = B_s(R_{i,j,s})$.

MSTd units compete with one another in recurrent networks [3, 7, 8] that implement divisive interactions. We define the activity of band-pass cells $P^{BP}_{i,j,s}$ using the following equation:

$$\begin{aligned} \frac{dP^{BP}_{i,j,s}}{dt} = &-\alpha^{MSTd} P^{BP}_{i,j,s} + (1 - P^{BP}_{i,j,s})(I^{BP}_{i,j,s} + Z(P^{BP}_{i,j,s}; \gamma^{MSTd}, \Gamma^{MSTd})) \\ &- P^{BP}_{i,j,s}(\sum_{n \neq i} \sum_{m \neq j} \sum_{o \neq s} Z(P^{BP}_{n,m,o}; \gamma^{MSTd}, \Gamma^{MSTd})) \end{aligned} \tag{21}$$

where α^{MSTd} defines the passive decay rate of each unit, $I^{BP}_{i,j,s}$ refers to the input signal, and $Z(P_{i,j,s}; \gamma^{MSTd}, \Gamma^{MSTd})$ is the on-center/off-surround recurrent feedback

MSTd units send one another. The last term involving the three summands means that each unit receives inhibition from units that either have a different receptive field position (n, m), or speed tuning (o). The recurrent feedback is defined by the sigmoid function

$$Z(w;\gamma,\Gamma) = \frac{(w-\Gamma)^2}{\gamma^2 + (w-\Gamma)^2} \tag{22}$$

where w is the activity of the unit sending the feedback signal, γ defines the inflection point of the sigmoid, and Γ represents the activity threshold that must be overcome in order to send the feedback signal. The sigmoid recurrent feedback function allows MSTd units to compete with one another to resolve winners without necessarily eliminating all the other weaker activity across the network (i.e. soft winner-take-all) [9, 10].

Speed summating $P^{SS}_{i,j}$ and gradient $P^{GC}_{i,j}$ cells obey similar equations, except note the absence of speed specific subscripts s:

$$\begin{aligned}\frac{dP^{SS}_{i,j}}{dt} = -\alpha^{MSTd}P^{SS}_{i,j} &+ (1-P^{SS}_{i,j})(I^{SS}_{i,j} + Z(P^{SS}_{i,j};\gamma^{MSTd},\Gamma^{MSTd})) \\ &- P^{SS}_{i,j}(\sum_{n\neq i}\sum_{m\neq j} Z(P^{SS}_{n,m};\gamma^{MSTd},\Gamma^{MSTd}))\end{aligned} \tag{23}$$

$$\begin{aligned}\frac{dP^{GC}_{i,j}}{dt} = -\alpha^{MSTd}P^{GC}_{i,j} &+ (1-P^{GC}_{i,j})(I^{GC}_{i,j} + Z(P^{GC}_{i,j};\gamma^{MSTd},\Gamma^{MSTd})) \\ &- P^{GC}_{i,j}(\sum_{n\neq i}\sum_{m\neq j} Z(P^{GC}_{n,m};\gamma^{MSTd},\Gamma^{MSTd}))\end{aligned} \tag{24}$$

Feedback $(MSTd - MT^-, MSTd - MSTv)$

MSTd units send feedback to suppress cells in MT^- and MSTv tuned to directions and speeds that are locally consistent with the global pattern preferred by the most active MSTd cell. The feedback described here implements the tuning curve $W(w;\mu,\sigma)$ introduced above (Eq. 4; see General Methods and Figure 3 for intuition). We evaluate the feedback tuning curve separately for speed and direction. For example, to resolve the direction component of the feedback, we evaluate $W(\theta_d;\chi_{i^*,j^*,x,y},\sigma^{MSTd,FB}_{dir})$ where θ_d represents the direction preference of the targeted MT units, $\chi_{i^*,j^*,x,y}$ (Eq. 12) is the set of angles corresponding to the preferred optic flow pattern of the most active MSTd cell whose preferred singularity is positioned at (i^*, j^*), and $\sigma^{MSTd,FB}_{dir}$ controls the extent to which the feedback signal affects $MT^-/MSTv$ units with similar direction preferences. The maximum weight in Eq. 4 is 1 because only the most active MSTd unit tuned to each pattern type may send feedback (i.e. winner-take-all).

We compute the feedback from band-pass ($K^{BP}_{x,y,d,s}$), speed-summating ($K^{SS}_{x,y,d,s}$), and gradient($K^{GC}_{x,y,d,s}$) cells as follows:

$$K^{BP}_{x,y,d,s} = \left[W(\theta_d;\chi_{i^*,j^*,x,y},\sigma^{MSTd,FB}_{dir})\right]\left[W(s;s^*,\sigma^{MSTd,FB}_{spd})\right] \tag{25}$$

$$K^{SS}_{x,y,d,s} = W(\theta_d;\chi_{i^*,j^*,x,y},\sigma^{MSTd,FB}_{dir}) \tag{26}$$

$$K^{GC}_{x,y,d,s} = \left[W(\theta_d;\chi_{i^*,j^*,x,y},\sigma^{MSTd,FB}_{dir})\right]\left[W(s;\tilde{E}_{s,i,j,x,y},\sigma^{MSTd,FB}_{spd})\right] \tag{27}$$

where s^* indicates the preferred speed of the most active MSTd unit and $\tilde{E}_{s,i,j,x,y}$ is the gradient cell annular speed sectors (Eq. 17). Note from Eq. 26 that speed summating cells send equivalent feedback, regardless of the speed tuning of the recipient cell, while band-pass cells concentrate the feedback weight around recipient cells tuned to similar preferred speeds (Eq. 25). Eq. 27 indicates that gradient cells send the strongest feedback to units most similar to those that sent feedforward input.

The feedback signal that reaches recipient cells in $MT^-/MSTv$ is the sum of Eqs. 25- 27:

$$K^{MSTd}_{x,y,d,s} = \sum_n \omega_n H\left[\omega_n - \Gamma^{FB}\right] \vartheta_n e^{b^{MSTd}((x-i)^2+(y-j)^2)} \tag{28}$$

where $\omega = \left\{P^{BP}_{i^*,j^*,s^*}, P^{SS}_{i^*,j^*}, P^{GC}_{i^*,j^*}\right\}$ and $\vartheta = \left\{K^{BP}_{x,y,d,s}, K^{SS}_{x,y,d,s}, K^{GC}_{x,y,d,s}\right\}$, $H\left[\cdot\right]$ represents the Heaviside step function, Γ^{FB} indicates the activity threshold that each type of MSTd unit must exceed to send feedback, and the exponential function increases feedback weights to $MT^-/MSTv$ units that have receptive fields at eccentricities far from the preferred singularity position (distance-dependent weighting).

The model proposes that the feedback to $MT^-/MSTv$ exerts a net inhibitory effect, which may or may not originate from inhibitory neurons in MSTd. If the feedback circuit follows the laminar anatomy common to other visual areas (e.g. between V1 and MT) [11–13], feedback projections from MSTd may be excitatory and target excitatory neurons in MT/MSTv. Within this canonical circuit, excitatory MSTd feedback projections may target several MT layers, including layer 6 [12], and neurons therein may project to inhibitory interneurons [14] as well as those involved in feedforward processing [15] within layer 4. Neurons in layer 4 project to layer 2/3 [16], which contains neurons with excitatory and inhibitory surrounds (MT^+ and MT^- cells) [17]. Thus, even though the MSTd–MT feedback projections may be excitatory, they may exert inhibitory effects on MT^- cells through local inhibitory populations distributed throughout MT, consistent with the structure of the model. Modeling these indirect pathways in detail from MSTd to MT^-/MSTv extends beyond the aims of the present paper, so we use inhibitory feedback as a simplification.

MT Layer 2-3 (MT^-)

MT^- units perform an on-center/off-surround integration of MT L4,6 signals. We obtain the center input by evaluating the 2D convolution function $C(\cdot)$ (Eq. 8): $C^-_{x,y,d,s,f} = C(M^{4,6}_{x,y,d,s,f}; x, y, \sigma^{MT^-,cent}, g^{MT^-,cent})$, which mimics the MT^+ center input (Eq. 9).

To resolve the spatial component of the inhibitory surround, we evaluate Eq. 8 at $S^{MT^-,1}_{x,y,d,s,f} = (C(M^{4,6}_{x,y,d,s,f}); x, y, \sigma^{MT^-,surr}, g^{MT^-,surr})$, where $\sigma^{MT^-,surr}$ controls the spatial extent of the integration with $\sigma^{MT^-,surr} > \sigma^{MT^-,cent}$, and $g^{MT^-,surr}$ is the inhibitory filter gain. We pass the output $S^{MT^-,1}_{x,y,d,s,f}$ of this convolution through a von Mises filter (Eq. 1) that defines the directional selectivity of the surround:

$$S^{MT^-,2}_{x,y,d,s,f} = g^-_{dir} V(S^{MT^-,1}_{x,y,d,s,f}, \theta_d, b^-_{dir}) \tag{29}$$

where b^-_{dir} controls the direction selectivity of the surround tuning and g^-_{dir} is the filter gain. The speed component of the inhibitory surround is computed according to:

$$S^{MT^-,3}_{x,y,d,s,f} = D(S^{MT^-,2}_{x,y,d,s,f}; s, \sigma^-_{spd}) \tag{30}$$

where σ^{-}_{spd} controls the speed selectivity of the surround and the function $D(\cdot)$ refers to the 1D Gaussian filter convolution (Eq. 19).

Model MT^{-} dynamics obey the following on-center/off-surround network:

$$\frac{dM^{-}_{x,y,d,s}}{dt} = -M^{-}_{x,y,d,s} + (1 - M^{-}_{x,y,d,s})C^{-}_{x,y,d,s,\lceil t \rceil} - (\beta^{-} + M^{-}_{x,y,d,s})(K^{MSTd}_{x,y,d,s} + S^{MT^{-},3}_{x,y,d,s,\lceil t \rceil}) \tag{31}$$

where β^{-} specifies the hyperpolarizing lower bound of each unit's activation when suppressed and $K^{MSTd}_{x,y,d,s}$ refers to the feedback signal from MSTd (Eq. 28). The inhibitory term in Eq. 31 exerts a divisive effect when β^{-} is small and a joint subtractive-and-divisive effect otherwise.

MSTv

MSTv units perform an on-center/off-surround integration of signals from MT^{-}, much in the same way MT^{-} integrates feedforward signals from MT L4,6. Aside from larger receptive fields, the main difference is that MSTv cells respond proportionally to the average speed, like speed summating cells in MSTd. To model this, we perform a spatial integration of rectified MT^{-} signals, weighted by their speed in both center and surround:

$$C^{v}_{x,y,d} = (C(U(\lceil M^{-}_{x,y,d,s} \rceil^{+})); x, y, \sigma^{v,cent}, g^{v,cent}) \tag{32}$$

$$S^{v,1}_{x,y,d} = (C(U(\lceil M^{-}_{x,y,d,s} \rceil^{+})); x, y, \sigma^{v,surr}, g^{v,surr}) \tag{33}$$

where $U(\cdot)$ is the weighted sum defined by Eq. 2 and $\lceil \cdot \rceil^{+}$ indicates rectification. We filtered the surround output $S^{v,1}_{x,y,d}$ by the von Mises filter V to establish direction selectivity:

$$S^{v-,2}_{x,y,d} = g^{v}_{dir} \sum_{n=1}^{\hat{d}} V(\theta_n, \theta_d, b^{v}_{dir}) S^{v,1}_{x,y,n} \tag{34}$$

The following equation describes the network dynamics of MSTv units $P^{v}_{x,y,d}$ (c.f. [7]):

$$\frac{dP^{v}_{x,y,d}}{dt} = -P^{v}_{x,y,d} + (1 - P^{v}_{x,y,d})C^{v}_{x,y,d} - (\beta^{MSTv} + P^{v}_{x,y,d})(U(K^{MSTd}_{x,y,d,s}) + S^{v,2}_{x,y,d}) \tag{35}$$

Modifications for Simulation 3

Notationally, we use h to index horizontal relative disparities in MT, $\delta^{4,6}_{h}$ to refer to specific depths to which MT units are tuned (units: cm relative to fixation), k indexes the disparities to which MSTd units are tuned, and ϕ_k refers to specific MSTd disparities in categorical units (e.g. 'near', 'fixational', and 'far').

Model input

We expanded the input motion vectors $\vec{v}_{x,y,f}$ to include the depth of each point: $\vec{v}_{x,y,f} = (v_{dx,f}, v_{dy,f}, v_{h,f})$, where $v_{h,f}$ refers to the depth relative to fixation in units of cm. Negative values reflect closer depths and positive values reflect farther depths. The fixation depth is assumed to remain consistent in each simulation. We defined MT disparity units maximally sensitive to one of five disparities: two crossed (near), two uncrossed (far), and one at fixation. Unless otherwise specified, the relative depths that yield the maximum responses ($\delta^{4,6}_{h}$) differ by 15 cm increments.

MT L4,6

Each disparity tuned unit integrates the input depth signal with weights defined by $q^{4,6}G(v_{h,f}; \delta_h^{4,6}, \sigma_h^{4,6})$, where $G(\cdot)$ refers to the Gaussian function defined by Eq. 4, $\sigma_h^{4,6}$ controls the width of the disparity tuning curves, and $q^{4,6}$ represents the disparity gain to simulate the tendency for stereo stimuli to garner greater activation in MT [18, 19]. We configured $\sigma_h^{4,6}$ to decrease with depth (Eq. 36), which resulted in the tuning curves that decrease in amplitude with depth due to filter normalization, as depicted in Figure 2d.

$$\sigma_h^{4,6}(h; \sigma_0, \sigma_{dh}) = \sigma_0 - \sigma_0\sigma_{ddisp}(h-1) \tag{36}$$

We evaluate Eq. 36 with $\sigma_h^{4,6}(h; \sigma_{disp_0}^{4,6}, \sigma_{ddisp_0}^{4,6})$ to obtain this behavior.

As mentioned above, we multiplicatively combine the disparity filters with those tailored to direction and speed:

$$M_{x,y,d,s,h,t}^{4,6} = \left[V\left(\Theta_{x,y,f}; \theta_d, b^{4,6}\right)\right]\left[G\left(\rho_{x,y,f}; \rho_s^{4,6}, \sigma_s^{4,6}\right)\right]\left[q^{4,6}G(v_{h,f}; \delta_h^{4,6}, \sigma_h^{4,6})\right] \tag{37}$$

Disparity tuning $(MT^+ - MSTd)$

We collapsed MT^+ disparities into three categorical values for band-pass cells and speed summating cells: 'near', 'fixational', and 'far' (ϕ_k). We applied the Gaussian weighting function $G(h; \phi_k, \sigma_{disp}^{MSTd,FF})$ defined by Eq. 4 to perform the transformation, where $\sigma_{disp}^{MSTd,FF}$ controls the degree of pooling between similar MT^+ disparities to resolve those in MSTd. The mapping is realized by the following convolution:

$$Q(w_k) = q^{MSTd}\sum_h G(h; \phi_k, \sigma_h^{MSTd,FF})w_h \tag{38}$$

where q^{MSTd} is the disparity gain. The type of disparity tuning defined by Eq. 38 corresponds to *non-direction-dependent disparity (non-DDD) cells* that comprise most of the disparity tuned cells in MSTd (Yang et al., 2011).

For gradient cells, we created direction $T_{d,i,j,x,y}$ (Eq. 14) and speed $E_{s,i,j,x,y}$ (Eq. 18) templates as before, but now we weight each speed annulus inversely with respect to depth. For example, if $s = 1$ indicates a slow speed, $s = 5$ indicates a fast speed, $d = 1$ indicates a near disparity, and $d = 5$ indicates a far disparity, we weight the region corresponding to $s = 1$ with $d = 5$, $s = 2$ with $d = 4$, and so on. We achieved this mapping with the following Gaussian convolution:

$$J_{d,s,i,j,x,y} = D(E_{s,i,j,x,y}; \hat{h} - h, \sigma_{disp}^{grad}) \tag{39}$$

Network dynamics

We obtain the input for band-pass and summating cells by composing the direction and speed functions defined above with the $MT^+/MSTd$ disparity mapping: $I_{i,j,s,k}^{BP} = Q(B_s(R_{i,j,s}); \phi_k, \sigma_{disp}^{MSTd})$. To compute the gradient cell input $I_{i,j}^{GC}$, first we perform the convolution specified by Eq. 39 to derive $J_{d,s,i,j,x,y}$, second we substitute $J_{d,s,i,j,x,y}$ for $E_{s,i,j,x,y}$ when solving for $I_{i,j}^{GC}$ (Eq. 20), and last we sum over MT disparities.

The dynamical equations for all types of disparity tuned MSTd units are identical to those defined above, except for the extra disparity dimension (e.g. $P_{i,j,s,k}^{BP}$).

Disparity specific feedback $(MSTd - MT^{-}/MSTd - MSTv)$

Disparity specific feedback from MSTd to MT^{-} and MSTv follows the same overarching rule outlined in General Methods (see Figure 3b): near MSTd units maximally inhibit near $MT^{-}/MSTv$ units, fixational units maximally inhibit fixational $MT^{-}/MSTv$, and far units maximally inhibit far $MT^{-}/MSTv$. We implement this in band-pass and speed summating cells by multiplying Gaussian disparity weights to those that define existing direction and speed based projections. For example:

$$K^{BP}_{x,y,d,s,h} = \left[W(\theta_d; \chi_{i^*,j^*,x,y}, \sigma^{MSTd,FB}_{dir})\right] \left[W(s; s^*, \sigma^{MSTd,FB}_{spd})\right] \left[W(h; k^*, \sigma^{MSTd,FB}_{disp})\right] \tag{40}$$

where $\sigma^{MSTd,FB}_{disp}$ controls the extent to which feedback from MSTd units tuned to a particular disparity influences $MT^{-}/MSTv$ cells tuned to other disparities.

MT Layer 2-3 (MT^{-})

We implement MT^{-} units with disparity tuned surrounds, following the same pattern as direction and speed. After computing the direction component $S^{MT^{-},2}_{x,y,d,s,t}$ (Eq. 29) and speed component $S^{MT^{-},3}_{x,y,d,s,t}$ (Eq. 30) of the MT surround, we added an analogous stage for disparity:

$$S^{MT^{-},4}_{x,y,d,s,h,t} = D(S^{MT^{-},3}_{x,y,d,s,h,t}; h, \sigma^{-}_{disp}) \tag{41}$$

where σ^{-}_{disp} determines the disparity selectivity of the surround.

MSTv

We configured the MSTv surrounds as untuned for disparity — motion at any disparity inhibits MSTv units when it appears in the surround:

$$S^{v,4}_{x,y,d,h} = q^{MSTv} \sum_{p \neq h} S^{v,3}_{x,y,d,p} \tag{42}$$

where, q^{MSTv} denotes the surround disparity gain.

Tables

Parameter	Area	Purpose	Description	Value	Unit
$\hat{d}$	All	Direction	Number of direction tunings	24	Ordinal
θ	All	Direction	Directions to which units are tuned	[-180, ...,165]	°
$\hat{s}$	All	Speed	Number of speed tunings	5	Ordinal
$\hat{h}$	All*	Disparity	Number of disparity tunings	5	Ordinal
$\hat{k}$	MSTd	Disparity	Number of MSTd disparity tunings	3	Ordinal
ϕ^{MSTd}	MSTd	Disparity	MSTd disparities	-2,0,2	N/A
$\hat{x}$	All*	Spatial	Size of horizontal dimension	64	Pixels (°)
$\hat{y}$	All*	Spatial	Size of vertical dimension	64	Pixels (°)

$\hat{i}$	MSTd	Spatial	Number of evenly spaced horizontal singularities to which MSTd units are tuned	16	Ordinal
$\hat{j}$	MSTd	Spatial	Number of evenly spaced vertical singularities to which MSTd units are tuned	16	Ordinal
q^{MSTd}	MSTd	Disparity	Disparity dependent gain	2.0	N/A
σ^{grad}_{disp}	MSTd	Disparity	Gradient cell disparity tuning width	0.05	N/A
σ^{grad}_{spd}	MSTd	Speed	Gradient cell speed tuning width	0.05	N/A
$\sigma^{MSTd,FF}_{disp}$	MSTd	Disparity	MT+–MSTd disparity tuning width	0.75	N/A
$\sigma^{MSTd,FB}_{dir}$	MSTd	Direction	MSTd–MT^{-}/MSTv direction tuning width	4	$^\circ$
$\sigma^{MSTd,FB}_{spd}$	MSTd	Speed	MSTd–MT^{-}/MSTv speed tuning width	0.2	N/A
$\sigma^{MSTd,FB}_{disp}$	MSTd	Disparity	MSTd–MT^{-}/MSTv disparity tuning width	4	N/A
Γ^{FB}	MSTd	All	Feedback signal threshold	0.01	mean spk/sec
g^{MSTd}	MSTd	Spatial	Feedback gain	1.0	N/A
b^{MSTd}	MSTd	Spatial	Extent of MSTd distance-dependent spatial integration from the preferred singularity position	5×10^{-3}	Pixels ($^\circ$)
$\sigma^{v,cent}$	MSTv	Spatial	Center RF extent	0.5	Pixels ($^\circ$)
$g^{v,cent}$	MSTv	Spatial	Center RF gain	4	N/A
$\sigma^{v,surr}$	MSTv	Spatial	Surround RF extent	4	Pixels ($^\circ$)
$g^{v,surr}$	MSTv	Spatial	Surround RF gain	6	N/A
q^{MSTv}	MSTv	Disparity	Surround disparity gain	1.5	N/A
b^{v}_{dir}	MSTv	Direction	Tuning width	7	Pixels ($^\circ$)
g^{v}_{dir}	MSTv	Direction	Tuning gain	0.26	N/A
σ^{-}_{disp}	MT^{-}	Disparity	Surround disparity selectivity	1	N/A
$\sigma^{MT^{-},cent}$	MT^{-}	Spatial	Center RF extent	0.5	Pixels ($^\circ$)
$g^{MT^{-},cent}$	MT^{-}	Spatial	Center RF gain	1.5	N/A
$\sigma^{MT^{-},surr}$	MT^{-}	Spatial	Surround RF extent	4	Pixels ($^\circ$)
$g^{MT^{-},surr}$	MT^{-}	Spatial	Surround RF gain	0.5	N/A
b^{-}_{dir}	MT^{-}	Direction	Tuning width	7	$^\circ$
g^{-}_{dir}	MT^{-}	Direction	Tuning gain	0.26	N/A
σ^{-}_{spd}	MT^{-}	Speed	Tuning width	1	N/A
$\sigma^{MT^{+},cent}$	MT^{+}	Spatial	Tuning width	3	Pixels ($^\circ$)
$b^{4,6}$	$MTL4,6$	Direction	Tuning width	4	$^\circ$
$\sigma^{4,6}_{spd}$	$MTL4,6$	Speed	Tuning width	0.01	N/A

$\sigma_{ds}^{4,6}$	$MTL4,6$	Speed	Speed-dependent increase in tuning width	0.1	N/A
$\sigma_{disp_0}^{4,6}$	$MTL4,6$	Disparity	Base tuning width	8	N/A
$\sigma_{ddisp_0}^{4,6}$	$MTL4,6$	Disparity	Disparity-dependent increase in tuning width	0.5	N/A

Table 1. Tuning curve parameter values used in simulations of neural model. $All*$ indicates that the parameter(s) apply to all areas of the model, except MSTd. Where applicable, we parameterized the model in pixel units for convenience for processing stimuli represented as a set of motion vectors in a sequence of digital images.

Parameter	Area	Description	Value	Unit
α^{MSTd}	MSTd	Decay rate	0.1	mean spk/sec
γ^{MSTd}	MSTd	Recurrent feedback sigmoid function infection point	0.12	mean spk/sec
Γ^{MSTd}	MSTd	Recurrent feedback signal threshold	0.28	mean spk/sec
β^{MSTv}	MSTv	Hyperpolarization activity lower bound	0.3	mean spk/sec
β^{-}	MT^{-}	Hyperpolarization activity lower bound	0.4	mean spk/sec
κ^{+}	MT^{+}	Depressive synapse recovery rate	10	mean spk/sec
τ^{+}	MT^{+}	Depressive synapse time constant	10	sec^{-1}

Table 2. Parameter values related to neural model dynamics. Spk denotes spike and mean spk/sec are in normalized units.

References

1. Browning NA, Grossberg S, Mingolla E. Cortical dynamics of navigation and steering in natural scenes: Motion-based object segmentation, heading, and obstacle avoidance. Neural Netw. 2009;22(10):1383–1398.

2. Browning NA, Grossberg S, Mingolla E. A neural model of how the brain computes heading from optic flow in realistic scenes. Cogn Psychol. 2009;59(4):320–356.

3. Layton OW, Fajen BR. Competitive dynamics in MSTd: A mechanism for robust heading perception based on optic flow. PLoS computational biology. 2016;12(6):e1004942.

4. Layton OW, Fajen BR. A neural model of MST and MT explains perceived object motion during self-motion. J Neurosci. 2016;36(31):8093–8102.

5. Mineault PJ, Khawaja FA, Butts DA, Pack CC. Hierarchical processing of complex motion along the primate dorsal visual pathway. Proc Natl Acad Sci U S A. 2012;109(16):E972–80.

6. Grossberg S. Intracellular mechanisms of adaptation and self-regulation in self-organizing networks: The role of chemical transducers. Bulletin of Mathematical Biology. 1980;42(3):365–396.

7. Layton OW, Mingolla E, Browning NA. A motion pooling model of visually guided navigation explains human behavior in the presence of independently moving objects. Journal of vision. 2012;12(1):20–20.
8. Layton OW, Fajen BR. Possible role for recurrent interactions between expansion and contraction cells in MSTd during self-motion perception in dynamic environments. J Vis. 2017;17(5):5.
9. Grossberg S. Contour enhancement, short term memory, and constancies in reverberating neural networks. Studies in Applied Mathematics. 1973;52:213–257.
10. Grossberg S. Recurrent neural networks. Scholarpedia. 2013;8(2):1888.
11. Felleman DJ, Van Essen DC. Distributed hierarchical processing in the primate cerebral cortex. Cerebral cortex (New York, NY: 1991). 1991;1:1–47.
12. Maunsell JH, Van Essen DC. The connections of the middle temporal visual area (MT) and their relationship to a cortical hierarchy in the macaque monkey. Journal of Neuroscience. 1983;3(12):2563–2586.
13. Bastos AM, Usrey WM, Adams RA, Mangun GR, Fries P, Friston KJ. Canonical microcircuits for predictive coding. Neuron. 2012;76(4):695–711.
14. Mcguire BA, Hornung JP, Gilbert CD, Wiesel TN. Patterns of synaptic input to layer 4 of cat striate cortex. Journal of Neuroscience. 1984;4(12):3021–3033.
15. Callaway EM. Local circuits in primary visual cortex of the macaque monkey. Annual review of neuroscience. 1998;21(1):47–74.
16. Wiser AK, Callaway EM. Contributions of individual layer 6 pyramidal neurons to local circuitry in macaque primary visual cortex. Journal of neuroscience. 1996;16(8):2724–2739.
17. Born RT. Center-surround interactions in the middle temporal visual area of the owl monkey. Journal of neurophysiology. 2000;84(5):2658–2669.
18. Nadler JW, Angelaki DE, Deangelis GC. A neural representation of depth from motion parallax in macaque visual cortex. Nature. 2008;452(7187):642.
19. Nadler JW, Barbash D, Kim HR, Shimpi S, Angelaki DE, Deangelis GC. Joint representation of depth from motion parallax and binocular disparity cues in macaque area MT. J Neurosci. 2013;33(35):14061–74, 14074a.
